# Immune-related long non-coding RNA signature identified prognosis and immunotherapeutic efficiency in bladder cancer (BLCA)

**DOI:** 10.1186/s12935-020-01362-0

**Published:** 2020-06-26

**Authors:** Rui Cao, Lushun Yuan, Bo Ma, Gang Wang, Ye Tian

**Affiliations:** 1 Department of Urology, Beijing Friendship Hospital, Capital Medical University, Beijing, 100050 China; 2 Department of Internal Medicine, Division of Nephrology, Leiden University Medical Center, 2333 ZA Leiden, The Netherlands; 3 Department of Stomatology, Beijing Shijitan Hospital, Capital Medical University, Beijing, 100038 China; 4 Department of Biological Repositories, Zhongnan Hospital of Wuhan University, Wuhan, 430071 China

**Keywords:** Bladder cancer, Immune infiltration, Immune checkpoint, LncRNA, TCGA

## Abstract

**Background:**

As bladder cancer was recognized to be immunogenic, dozens of studies have focused on immune biology of BLCA, but little is known about its relationship with the long non-coding RNAs (lncRNAs).

**Methods:**

LASSO Cox regression model was used to establish immune-related lncRNAs signature (IRLS) in BLCA. The immune infiltration landscape of BLCA was conducted via ssGSEA and immunotherapy response was calculated through TIDE algorithm.

**Results:**

A total of 82 immune-related lncRNAs were screened out according to spearman correlation analysis with the immune score (|R| > 0.4, p < 0.05). We selected 5 prognostic lncRNAs to construct immune-related lncRNAs signature (IRLS) through LASSO Cox regression analysis. Then we validated that 5 enrolled lncRNAs was downregulated in BLCA tissues and cells when compared with paracancerous tissues and normal bladder epithelium cell. The univariate and multivariate Cox regression analysis both demonstrated the IRLS was a robust independent prognostic factor in overall survival prediction with high accuracy. The GSVA and GSEA also suggested that the IRLS are involved in the immune-related biological processes and pathways which are very well known in the context of BLCA tumorigenesis. In addition, we found that IRLS is strikingly positive correlated with tumour microenvironment (TME) immune cells infiltration and expression of critical immune checkpoints, indicating that the poor prognosis might be caused partly by immunosuppressive TME. Finally, the results from the TIDE analysis revealed that IRLS could efficiently predict the clinical response of immunotherapy in BLCA.

**Conclusion:**

We have developed a novel IRLS, which have a latent prognostic value for BLCA patients and might facilitate personalized counselling for immunotherapy.

## Background

Bladder cancer (BLCA) is a disease within urinary tract of high malignancy, which has nearly 549,000 new cases and 200,000 deaths and ranks the 10th most common cancer in 2018 [1]. As identified as heterogeneous carcinoma, there are two major subtypes: non-muscle-invasive bladder cancer (NMIBC) and muscle-invasive bladder cancer (MIBC). NMIBC, which consist the majority of the BLCA, is not that fatal but have high potential to recur [2]. Intra-vesicular administration of chemotherapeutics and Bacillus Calmette-Guerin (BCG) was utilized to prevent recurrence and progression [3]. It is essential to note that BLCA was recognized to be immunogenic after the successful instillation-therapy of BCG which was conducted by Alvaro Morales in 1976 [4]. Moreover, the BCG was also identified as the second immunotherapy drug, which was approved by United States Food and Drug Administration (FDA), to inhibit the tumour growth, just behind the interferon-alpha (IFN-α) [5]. Although BCG is the first-line therapy for NMIBC patients, especially when contaminated with carcinoma in situ (CIS), not all the patients benefit from it and some responders will finally relapse and progress to the another subtype MIBC, which is more life-threatening and need systemic therapy [6, 7]. Recently, more and more study have focused on the onco-immunology and a lot of immune-checkpoint inhibitors (ICIs) were developed and showed a robust and durable responses in patients with various cancers [8, 9], including BLCA [10].

The development of transcriptome sequencing over the past decade have revealed that over 70% of the genome is transcribed into RNA, among them vast majority are non-coding RNAs (ncRNAs) [11, 12]. Long non-coding RNAs (lncRNAs) are a major type of ncRNAs with more than 200 nucleotides in length, which has been previously considered as ‘junk’ or ‘transcriptional noise’ for not capable of coding protein [13]. As the flourish of the study in lncRNAs, we have found that lncRNAs acted as a key regulator in a broad range of biological processes, including cell differentiation, proliferation [14]. Therefore, lncRNAs were further identified as the novel initiator and promoters for neoplasia by their specific expression and subcellular localization [15]. The expression of lncRNAs usually varied during the development process [16]. LncRNAs have exerted their role in tumorigenesis through regulation of genes associated with cell proliferation, differentiation and migration by epigenetic regulations, interference with the transcriptional machineries, inducing alternative splicing etc. [17]. There is growing evidence that lncRNAs function as potential biomarkers as well as therapeutic targets in many cancer types, especially in carcinoma in urinary tract [18–20]. A lot of studies found that urothelial carcinoma-associated (UCA1) lncRNA has been significantly up-regulated in BLCA compared with the paracancerous tissues. And UCA1 could influence the cisplatin/gemcitabine sensitivity through targeting CREB modulating miR-196a-5p in BLCA cells [21, 22]. Moreover, the increase expression of LncRNA TUG1 was reported to be related with poor overall survival (OS) in BLCA [23]. Moreover, recently some lncRNAs signatures have been identified to be prognostic, which might give us a chance to establish more accuracy biomarkers for BLCA [24, 25]. But the immune-related lncRNAs signature was not often investigated.

In the present study, we have established a 5 immune-related lncRNAs (AC005014.2, AC010503.4, AL450384.2, LINC00930 and SH3BP5-AS1) signature (IRLS) through LASSO Cox regression analysis. All selected 5 lncRNAs were downregulated in BLCA tissues and cells compared with pancancerous tissues and normal bladder epithelium cell. Then we found that the IRLS performed well in over survival (OS) prediction and acted as an independent prognostic factor via univariate and multivariate Cox regression analysis in BLCA. Thus, the annotation and function analyses also indicated that our model was involved in the immune-related response processes and pathways which play a vital role in BLCA tumorigenesis. In addition, we also found that the IRLS was highly correlated with the TME immune cells infiltration and ICIs immunotherapy response based on the TIDE algorithm. In summary, we have constructed a novel IRLS, which have a potential prognostic value for BLCA patients and might facilitate personalized counselling for immunotherapy.

## Materials and methods

### Ethical statement for human bladder tissue samples

As described previously by our group [26, 27], the MIBC tissues and paracancerous tissues (n = 20) used in this study were collected from patients with MIBC after radical resection at Zhongnan Hospital of Wuhan University, Wuhan, China. The tissue specimens were immediately stored in liquid nitrogen for total RNA isolation. Informed consent was provided by all subjects. All specimen collection and treatments were carried out in accordance with the approved guidelines according to the Ethics Committee at Zhongnan Hospital of Wuhan University (approval number: 2015029, Related file in Additional file 1: Ethics approval).

### Human bladder cancer cell lines

Human bladder cancer cell lines RT-4 (Cat. #TCHu226), 5637 (Cat. #TCHu1), T24 (Cat. #SCSP-536), UM-UC-3 (Cat. #TCHu217), J82 (Cat. #TCHu218), SCaBER (Cat. #TCHu239), SW780 (Cat. #TCHu219) and human immortalized normal urothelium cell line SV-HUC-1 (Cat. #TCHu169) were kindly provided by the Stem Cell Bank, Chinese Academy of Sciences in Shanghai, China. Another human bladder cancer cell line, EJ (Cat. #CL-0274), was purchased from the Procell Co., Ltd. in Wuhan, China. The T24 and RT-4 cells were maintained in McCoy’s 5 A Medium (Gibco, China). The UM-UC-3 and J82 cells were maintained in MEM medium (Gibco, China). The 5637, SCaBER, and EJ cells were maintained in RPMI-1640 medium (Gibco, China). The SV-HUC-1 cell was maintained in F12K medium (Gibco, China). All medium was supplemented with 1% penicillin G sodium/streptomycin sulfate and 10% fetal bovine serum (FBS) (Gibco, Australia). All cells were grown in a humidified atmosphere consisting of 5% CO_2_ and 95% air at 37 ℃.

### Total RNA isolation from bladder tissues and BCa cells

Total RNA from cells and tissues were isolated with the Qiagen RNeasy Mini Kit (Cat. #74101, Qiagen, Germany), and QIAshredder from Qiagen (Cat. #79654, Qiagen, Germany) using a centrifuge (Cat. #5424, Eppendorf, Germany) according to the manufacturer’s protocol. DNase I (RNase-Free DNase Set, Cat. #79254, Qiagen, Germany) was used to remove the contamination of gDNA in each RNA sample. The quantity of isolated RNA was measured by NanoPhotometer (Cat. #N60, Implen, Germany).

### Reverse transcription and quantitative real time PCR (qRT-PCR)

First-strand cDNA was synthesized by ReverTraAce qPCR RT Kit (Toyobo, China) using 1 µg of total RNA isolated from tissues or cells. Each reaction of real-time polymerase chain reactions (PCR) was conducted with iQTM SYBR^®^ Green Supermix (Bio-Rad, China) in a final volume of 20 µl using 1 µg of cDNA. All primers were tested for optimal annealing temperatures and PCR conditions were optimized with gradient PCRs on an iCycler (Cat. #CFX Connect, Bio-Rad, USA). Primer sequences and annealing temperatures are summarized in Additional file 2: Table S1. Values were normalized for amplified *GAPDH* alleles. Relative gene abundance = 2^−∆∆ct^, ∆ct = ct_*target gene*_−ct_*GAPDH*_, for cells ∆∆ct = ∆ct_*BLCA cells*_−∆ct_*SV*-*HUC*-*1 cell*_, for tissues ∆∆ct = ∆ct_*BCa tissues*_−∆ct_*paracancerous tissues*_ (ct = threshold cycle).

### Data collection and processing

The public available transcriptomic cohort for BLCA with full clinical information from the The Cancer Genome Atlas (TCGA) was downloaded from the UCSC Xena (GDC hub) (https://tcga.xenahubs.net). The samples without complete overall survival (OS) information were not enrolled for further evaluation. The transcripts per million reads (TPM) will be represented as the gene expression of RNA instead of the fragments per kilobase of exon per million reads mapped (FPKM), which was obtained from the TCGA-BLCA RNA-sequencing data. The gene symbol was annotated at the highest expression according to theENSEMBL ID. Finally TCGA-BLCA cohort consisting of 403 samples was defined as an entire set, which was then randomly separated into training and testing cohorts at cut-off 7:3. Detailed information of clinicopathological characteristics in TCGA-BLCA cohorts could be found in our previous study [28]. Data were analysed with the R (version 3.5.2) and R Bioconductor packages.

### Identification of immune-related LncRNAs

**Fig. 1:**
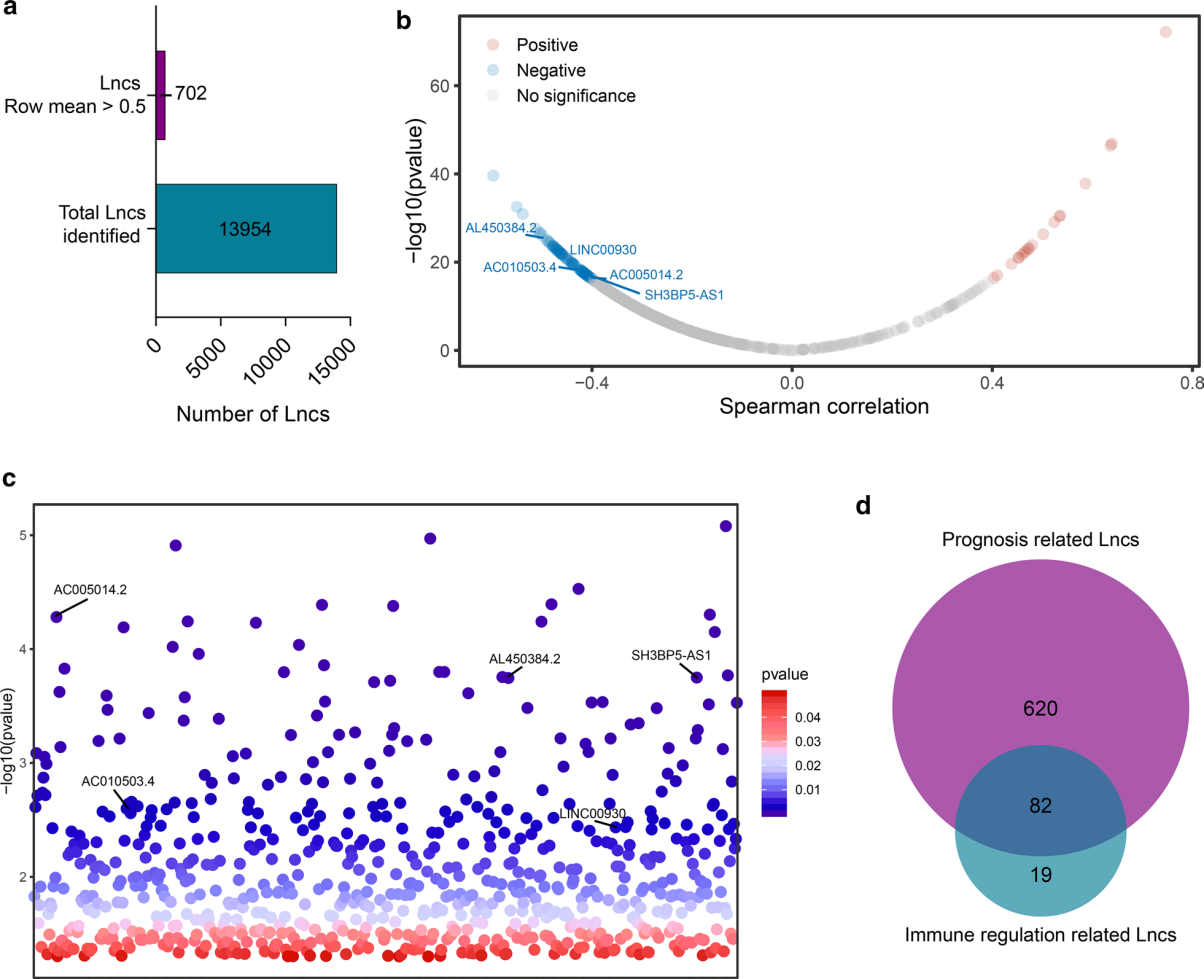
Identification of prognostic immune-related candidate lncRNAs in TCGA-BLCA cohort. **a** Histogram indicated the total annotated lncRNAs and low expression filtered lncRNAs **b** The dot plot demonstrated the correlation between lncRNAs and immune score through spearman correlation analysis. The red indicated positive correlation and the blue indicated negative correlation. The selected lncRNAs with IRLS were listed. The cut-off was defined as |R| > 0.4, p < 0.05. **c** The dot plot of prognostic lncRNAs. The selected lncRNAs with IRLS were listed. **d** Venn plot for prognostic lncRNAs and immune-related lncRNAs

The immune-related genes were obtained from gene set M13664 (immune system process) and M19817 (immune response) in MSigDB of Broad Institute (http://software.broadinstitute.org/gsea/index.jsp) [29, 30]. The single-sample gene set enrichment analysis (ssGSEA) was used to calculate the immune scores of each sample in TCGA-BLCA cohort [31, 32]. The low expression lncRNAs with rowmeans ≤ 0.5 were removed from the further study. Then the immune-related lncRNAs were identified for high correlation with the immune score (|R| > 0.4, p < 0.05) based on spearman correlation analysis. Kaplan–Meier (KM) survival analyses were utilized to screen out the prognosis related lncRNAs (p < 0.05). After merging the immune-related and prognosis related lncRNAs, the remained selected lncRNAs were considered to be immune-related candidate lncRNAs. The process of the selection was shown in Fig. 1.

### Establishment and validation of prognostic IRLS

The selected immune-related candidate lncRNAs mentioned above were submitted to LASSO Cox regression analysis based on package “*glmnet*” in R for building an optimal prognostic immune-related lncRNAs signature (IRLS) for BLCA [33]. Then IRLS risk-score for each patient in TCGA-BLCA cohort was defined as the relative expression of each lncRNA and its associated Cox coefficient. The formula for ${\text{IRLS risk}} - {\text{score}}\, = \,\sum_{i = 1}^{n} \;({\text{coef}}_{\text{i}} \, \times \,{\text{Expr}}_{\text{i}} )$, where Expr_i_ is the relative expression of lncRNA in the signature for patient i, coef_i_ is the LASSO Cox coefficient of the lncRNA i. Furthermore, all patients were stratified into the high-risk and low-risk groups according to the IRLS risk-score. KM survival analyses were used to evaluate the OS between IRLS high/low risk patients and indicated stratified clinical features by using package “*survminer*” in R. The time-dependent receiver operating characteristic (ROC) curves were utilized to assess the prediction accuracy in prognosis prediction of IRLS and the area under curve (AUC) for 1-year, 3-year and 5-year OS was measured using package “*survivalROC*” in R [34].

### Correlation between IRLS and clinicopathological characteristics

The correlation between IRLS risk-scores with corresponding clinicopathological characteristics, including age, grade, histological subtype, pathological T stage, pathological N stage, pathological M stage, pathological tumour stage, lymphovascular invasion status and number of positive lymphonodes by hematoxylin and eosin (HE), was measured by *t* test or one-way ANOVA test and shown by box plot. Furthermore, cluster heat map were conducted on the immune-related lncRNAs and infiltration of each immune cell types according to IRLS risk-level by utilizing the package “*pheatmap*” in R. The correlation between clinicopathological characteristics with IRLS risk-level was calculated by *χ*^2^ test. *p < 0.05, **p < 0.01, ***p < 0.001.

### Functional and annotation analyses

The Hallmark gene sets, which were also downloaded from the MSigDB of Broad Institute (http://software.broadinstitute.org/gsea/index.jsp) [35], were used to analyse the change of pathway enrichment based on IRLS through package “*GSVA*” in R [36]. The significantly enriched pathways in Hallmark gene sets were identified with a threshold of p < 0.05 and t value > 2. Furthermore, ssGSEA score were calculated for the significantly changed gene sets, then the cluster heat map and correlation between IRLS and enriched pathways was measured. Moreover, GSEA was conducted to assess the influence of IRLS on M13664 (immune system process) and M19817 (immune response) gene set via package “*clusterProfiler*” in R to show the common GSEA plot [37].

### Construction of a predictive nomogram

The IRLS and clinical features were merged to find independent prognostic factors through univariate and multivariate Cox regression analysis and visualized through package “*forestplot*” in R. Then the nomogram integrating with selected independent prognostic factors was established through package “*rms*”, “*nomogramEx*” and “*regplot*” in R [38]. Furthermore, decision curve analysis (DCA) and calibration curves were used to see whether our nomogram was useful as the ideal model.

### Estimation of TME immune infiltrating and immune-checkpoint inhibitors (ICIs) response

The gene set which could represent different infiltrating immune cell types was obtained from Bindea et al. [39]. Then ssGSEA was utilized to calculate the abundance of immune cell according to the expression of reference gene within the gene set from transcriptomic data. The 24 types of immune cells were enrolled in our study, including innate immune cells (dendritic cells [DCs], immature DCs [iDCs], activated DCs [aDCs], plasmacytoid DC [pDCs], eosinophils, mast cells, macrophages, natural killer cells [NKs], NK CD56dim cells, NK CD56bright cells, and neutrophils) and adaptive immune cells (B cells, T cells, T helper cells, T helper 1 [Th1], Th2, T gamma delta [Tγδ], CD8 + T, T central memory [Tcm], T effector memory [Tem], T follicular helper [Tfh] cells, T helper 17 (Th17) cells, regulatory T (Treg) cells and cytotoxic cells). The immune scores, stromal scores and estimate scores from each sample were calculated by applying the “Estimation of STromal and Immune cells in MAlignant Tumours using Expression data” (ESTIMATE) algorithm [31]. Moreover, the immune-checkpoint inhibitors (ICIs) response was assessed through Tumour Immune Dysfunction and Exclusion (TIDE) algorithm according to the suggestion of Hoshida et al. [40].

### Statistical analyses

Statistical significance for variables between two groups or more than two groups was estimated by unpaired Student t tests or one-way ANOVA tests respectively. The *χ*^*2*^ test was applied to analyse the correlation between IRLS risk-level and clinicopathological characteristics. Kaplan–Meier (KM) survival curves and log-rank test were used to assess differences in survival between different groups using the package “*survminer*” in R. The spearman correlation analyses were used to detect the correlation between two parameters. Two-sided Fisher’s exact tests were used to evaluate the efficacy of ICIs between different groups. Univariate and Multivariate Cox proportional-hazard models were utilized to assess the hazard ratios of variables and identify independent prognostic factors. Nomogram, calibration curve and DCA were constructed according to *Iasonos*’ suggestion [38]. A time-dependent receiver operating characteristic curve (ROC) analyses were used to compare the predictive accuracy. All statistical analyses were performed with R software 3.5.3. Statistical significance was set at probability values of p < 0.05.

## Results

### Identification of prognostic and immune-related LncRNAs

A flow diagram and design of the study can be seen in Additional file 3: Figure S1. The transcriptomic data of 403 patients with full clinical information were retrieved from the TCGA-BLCA cohort. Then 13954 lncRNAs were identified by mapping from the ENSEMBL ID (Fig. 1a). After filtering the low expression lncRNAs, the 702 prognostic related lncRNAs were screened out through Kaplan–Meier (KM) survival analyses (Fig. 1c). Furthermore, the immune scores in each sample were calculated via ssGSEA according to the reference of the M13664 (immune system process) and M19817 (immune response) gene sets. After measured with spearman correlation analyses, 101 lncRNAs were recognized as immune-related lncRNAs (|R| > 0.4, p < 0.05) (Fig. 1b). At last, we have got 82 prognostic immune-related candidate lncRNAs for further research (Fig. 1d).

### Establishment of immune-related LncRNAs signature (IRLS)

**Fig. 2:**
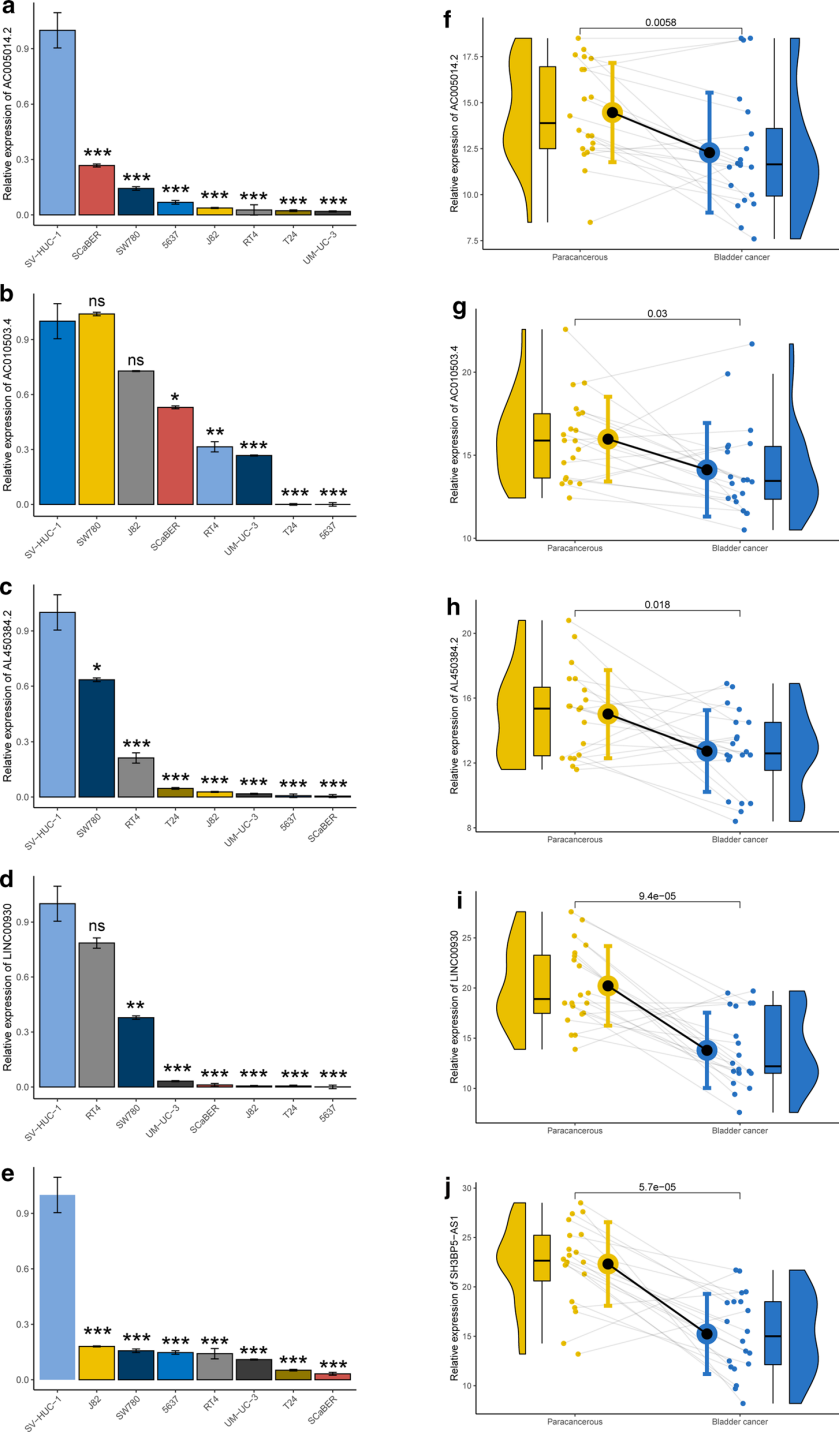
Elevated expression of 5 selected lncRNAs in paracancerous tissues and normal urothelium cell compared with BLCA tissues and cells. **a**–**e** qRT-PCR analysis indicates the transcriptional level of AC005014.2 (**a**), AC010503.4 (**b**), AL450384.2 (**c**), LINC00930 (**d**) and SH3BP5-AS1 (**e**) in distinct malignancy BLCA cells (EJ, UM-UC-3, T24, 5637, J82, SCaBER and RT-4) and normal immortalized urothelium cell SV-HUC-1. The GAPDH allele is used as a loading control. The gene expression of SV-HUC-1 cells was characterized as the control to compare the relative gene expression in each cell, *p < 0.05, **p < 0.01, ***p < 0.001, ns: p > 0.05. **f–j**) qRT-PCR analysis exhibits the expression of AC005014.2 (**f**), AC010503.4 (**g**), AL450384.2 (**h**), LINC00930 (**i**) and SH3BP5-AS1 (**j**) at the transcription level in BLCA tissues compared with that in paired paracancerous tissues. The GAPDH allele is used as an internal control. The difference between BLCA and paired pancancerous tissues were measured with Student t tests. The p value was indicated in the figures

The entire TCGA-BLCA cohort was randomly divided into training and testing cohorts at the cut-off 7:3. As 82 prognostic immune-related candidate lncRNAs might display the similar function and biological process, the LASSO Cox regression analysis was used for dimension reduction. Then we constructed an immune-related lncRNAs signature (IRLS) consisting of 5 lncRNAs, which could predict the overall survival (OS) in TCGA-BLCA training cohort, and the formula for IRLS risk-score was calculated as follows: expression of AC005014.2 * (− 0.05409) + expression of AC010503.4 * (− 0.0002344) + expression of AL450384.2 * (− 0.01291) + expression of LINC00930 * (− 0.008834) + expression of SH3BP5-AS1 * (− 0.04496) (Additional file 4: Figure S2). The KM log-rank test survival analyses demonstrated that all five lncRNAs within IRLS could predict the OS of BLCA effectively and acted as protective factors for BLCA patients (Additional file 5: Figure S3). Then we measured the relative expression of AC005014.2, AC010503.4, AL450384.2, LINC00930 and SH3BP5-AS1 in BLCA tissues and cells. Surprisingly, we found that relative expression of 5 selected lncRNAs were upregulated in SV-HUC-1 (human immortalized normal urothelium cell line) when compared with almost all BLCA cell linses (Fig. 2a–e). Moreover, qRT-PCR results from our own specimens verified a significant overexpression of 5 lncRNAs in the paracancerous tissues compared with that in the paired BLCA tissues, which was in accordance with the results in cells (n = 20, Fig. 2f–j, p < 0.05).

**Fig. 3:**
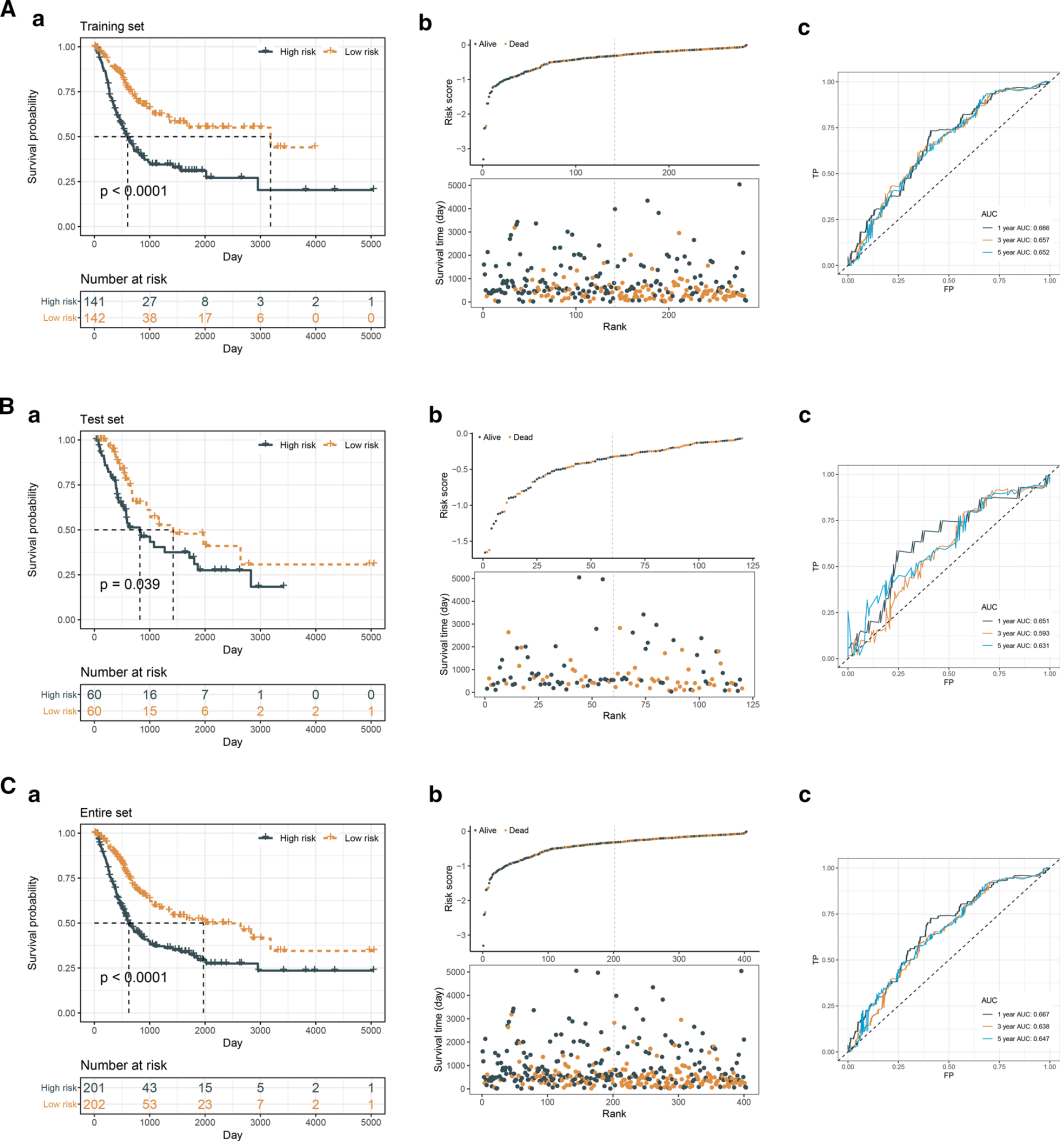
Immune-related lncRNAs signature (IRLS) is a prognostic biomarker for overall survival (OS) in TCGA-BLCA cohort. (**A**–**C** a**–**c) KM survival, risk score and time-dependent ROC curves of OS according to IRLS groups in TCGA-BLCA training (**A**), testing (**B**) and entire (**C**) cohort. The entire cohort was divided into the training and testing cohorts at 7:3 cut-off. The cohorts were all stratified at median cut-off of the IRLS risk-scores to form IRLS high-risk and low-risk groups. The AUC was assessed at 1, 3 and 5 years

Then we stratified the patients into low-risk or high-risk groups at median cut-off based on the IRLS risk-scores. KM survival curves indicated that the IRLS low-risk patients lived longer than IRLS high-risk patients in the training cohort (p < 0.0001) (Fig. 3A a, b). Therefore, time-dependent ROC analysis showed an appropriate accuracy of IRLS in predicting OS in training cohort and area under the ROC curve (AUC) was 0.666 at 1 year, 0.657 at 3 years and 0.652 at 5 years (Fig. 3A c). Moreover, these were further validated in the testing and entire cohort in order to assess the robust prediction value of IRLS. We found that the results in testing and entire cohort were consistence with the outcome in training cohort, indicating all the high-risk patients were associated with poorer prognosis (Fig. 3B a-b and Fig. 3C a-b). In the testing cohort, the significant prognostic value was p = 0.039 and AUC with 1-, 3- and 5-years were 0.651, 0.593, 0.631, respectively (Fig. 3B c). In the entire cohort, the significant prognostic value was p < 0.0001 and AUC with 1-, 3- and 5-year were 0.667, 0.638, 0.647, respectively (Fig. 3C c).

### Correlation between IRLS and clinicopathological characteristics

**Fig. 4:**
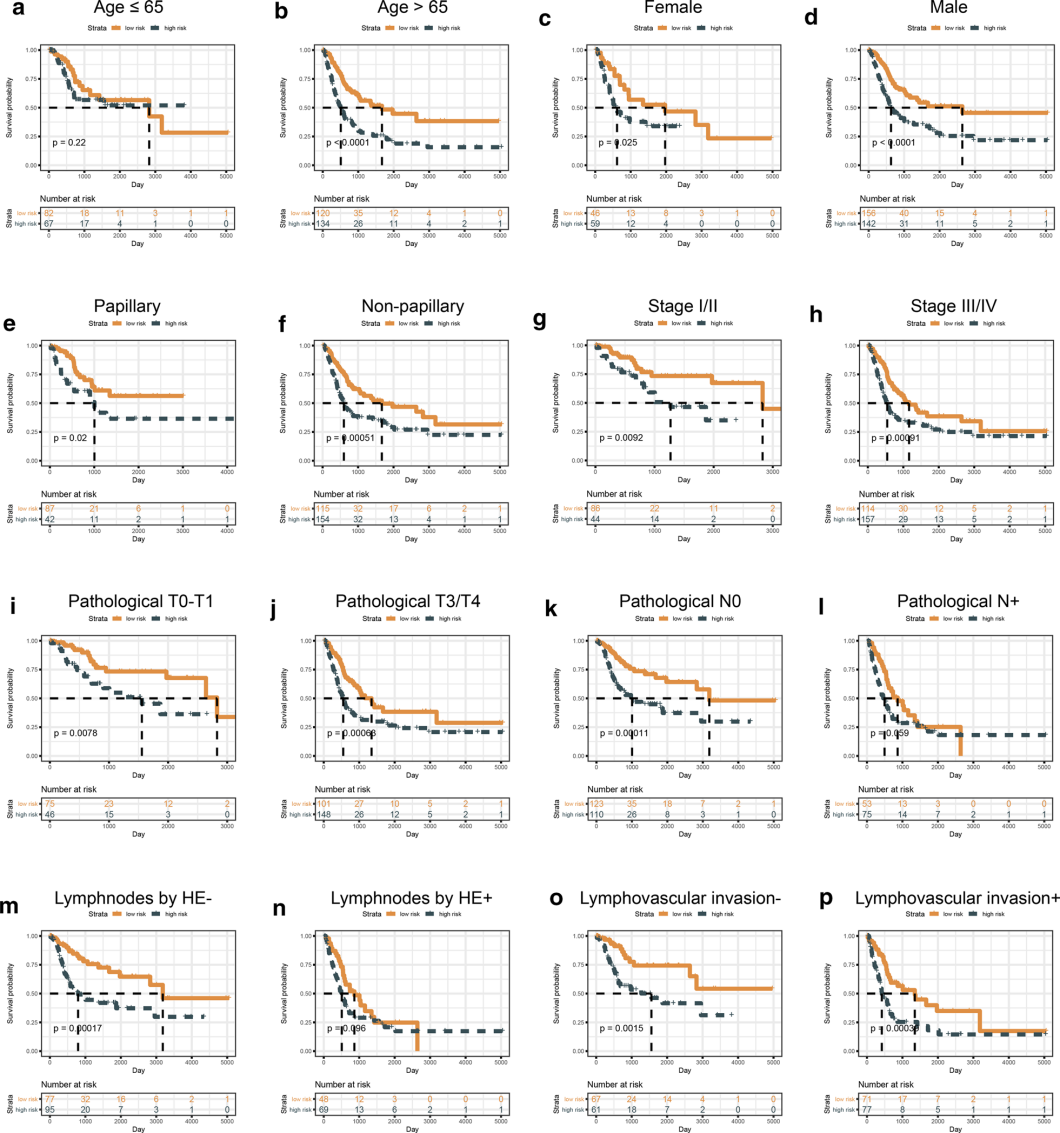
KM survival stratification analyses in TCGA-BLCA cohort. **a** Age ≤ 65 years; **b** Age > 65 years; **c** Female; **d** Male; **e** Papillary; **f** Non‐papillary; **g** Stage I/II; **h** Stage III/IV; **i** Pathology T0‐T2; **j** Pathology T3‐T4; **k** Pathology N0; **l** Pathology N + ; **m** Number of positive lymph nodes by HE −; **n** Number of positive lymph nodes by HE + ; **o** Lymphovascular invasion −; **p** Lymphovascular invasion +

In order to figure out the role of IRLS in BLCA progression, the correlations between IRLS and clinical features was investigated. The boxplot showed that patients of IRLS high-risk were more likely to be the elder, non-papillary, high TNM stage and grade patients, which indicated of the high correlation of IRLS with tumour malignancy (Additional file 6: Figure S4). And the cluster heat map revealed that all lncRNAs within IRLS were downregulated in high-risk group (Fig. 7a). The stratification survival analyses were utilized to see whether the IRLS could apply in different clinicopathological characteristics. Thus, we found that IRLS could efficiently predict the OS in almost all the subgroups from the different clinical features (Fig. 4).

### Identification of IRLS related functional annotation

**Fig. 5:**
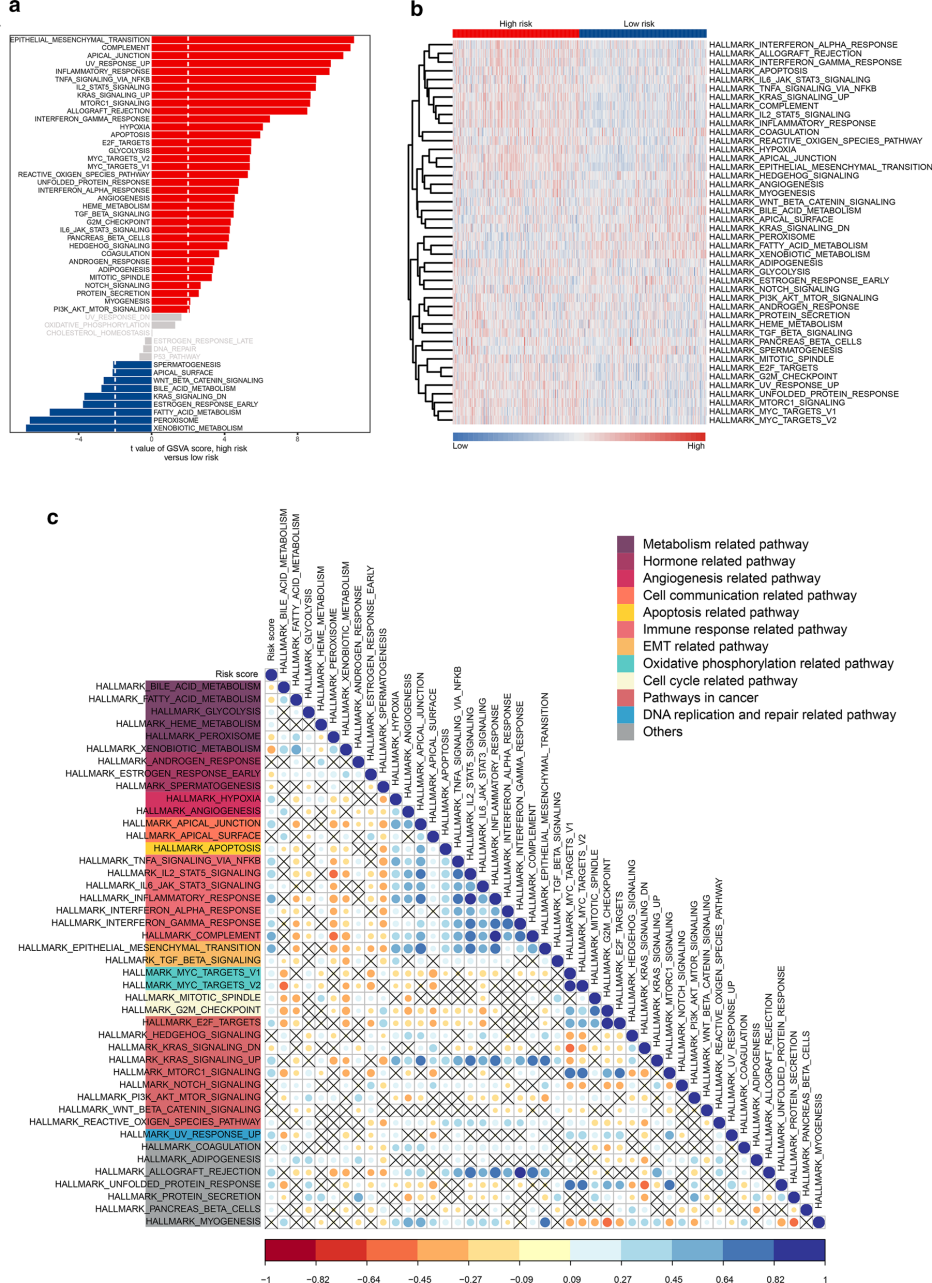
The GSVA of hallmark gene sets in TCGA-BLCA cohort. **a** The bar plot showed the results of GSVA in the TCGA-BLCA. **b** The cluster heat map of the hallmark gene sets. The red indicated IRLS high-risk samples and blue indicated IRLS low-risk samples. **c** Correlation matrix of IRLS values and the activation levels of hallmark gene sets. The similar functional gene sets were identified as common signalling pathways with the same colour described in legends. Shading colour represents the value of corresponding correlation coefficients and nonsignificant correlations are denoted by “X”; blue indicated positive correlation and red indicated negative correlation

The GSVA was used to figure out the dynamics of biological pathways and processes according to the IRLS based on the Hallmark gene sets. The detail results of GSVA could be found in Additional file 7: Table S2. We found that the IRLS high-risk group were enriched in angiogenesis, immune response, epithelial-mesenchymal transition (EMT) related pathway, etc. which played a vital role in tumorigenesis. Meanwhile, the metabolism related pathway was enriched in IRLS low-risk group (Fig. 5a, b). In order to comprehensive unfold the functional annotation, the correlation analysis was conducted to the common activated/suppressed gene sets (Additional file 8: Table S3). The results showed that the gene sets within immune response related pathway were significantly correlated with each other when compared with other pathways. And the IRLS risk-score was highly positive associated with all the immune related gene sets (Fig. 5c). Moreover, the GSEA also revealed that immune response and immune system process gene sets, which were used to represent the immune score in each sample, were high enriched in the IRLS low-risk group (Additional file 9: Figure S5). All these demonstrated the IRLS could be a good model to represent for immune response status, which is so important for BLCA.

### The IRLS was an independent prognostic factor in BLCA

**Fig. 6:**
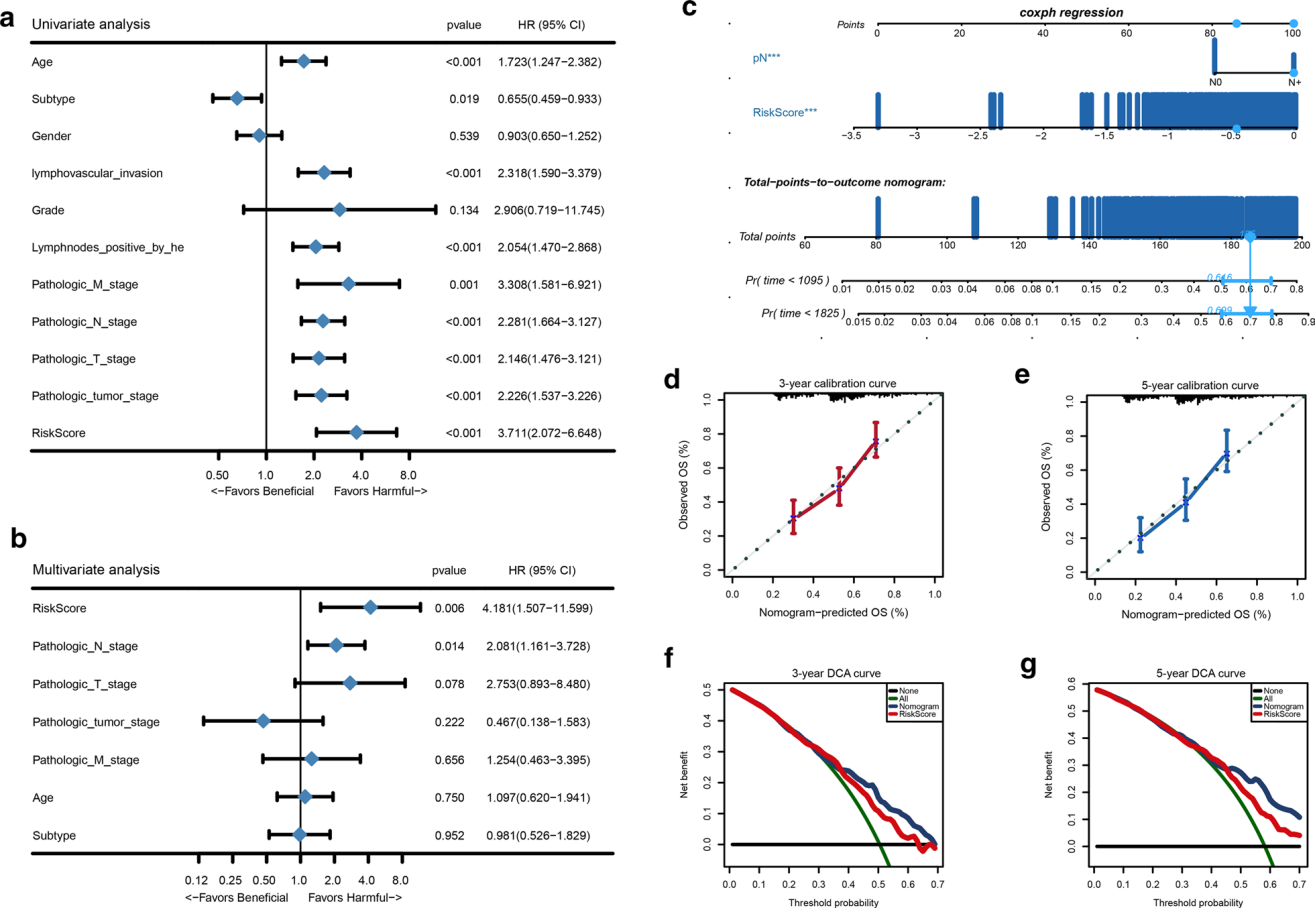
IRLS is an independent prognosis factor in the nomogram. **a, b** Forest plot summary of the univariate and multivariable Cox analyses of the IRLS and clinicopathological characteristics. The blue diamond squares on the transverse lines indicate the HR, and the black transverse lines indicate the 95% CI. **c** Nomograms integrating the IRLS and pathology N stage for predicting the probability of patient mortality at 3- or 5-year OS. **d, e** Calibration curves of the nomogram for predicting the survival outcomes at 3-, and 5-years.The 45-degree line represents the ideal prediction. **f–g** Decision curve analyses (DCA) curve of the nomograms for 3-year and 5-year OS

Among all the clinical features, we wanted to make it clear whether IRLS, which could represent the immune response status, was an independent prognostic factor in BLCA. By integrating all the clinicopathological characteristics with IRLS, univariate Cox regression analysis demonstrated that all except gender and tumour grade were responsible for OS in BLCA (Fig. 6a). Then the multivariate Cox regression analysis confirmed that IRLS and pathological N stage were the only two independent factors for predicting the prognosis of BLCA patients (Fig. 6b). Then we established a nomogram consisting of these two prognostic variates, which could predict the mortality of BLCA patients via the quantitative scoring method (Fig. 6c). According to the nomogram, every patient will get a total point from each prognostic parameter. The higher point indicated the higher mortality the patients were. Furthermore, calibration curves demonstrated that the prediction accuracy of our model was similar to the ideal model (Fig. 6d, e). The DCA revealed that the nomogram have an advantage of IRLS alone and displaced a high potential for clinical utility (Fig. 6f, g).

### The landscape of TME immune cells infiltration in BLCA

**Fig. 7:**
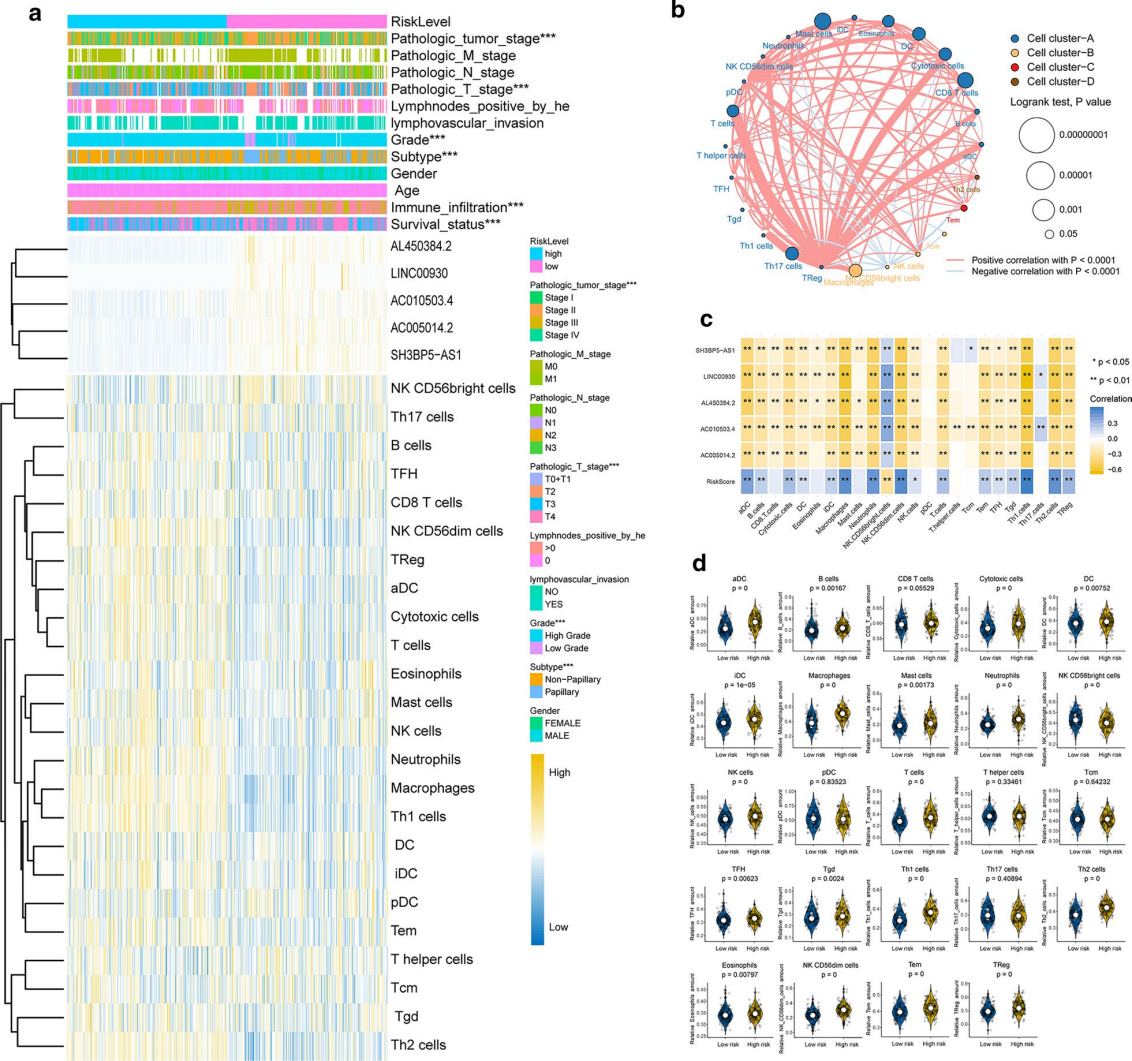
The IRLS is associated with TME immune cells infiltration. **a** Cluster heat map of immune-related lncRNAs and 24 types of immune cells stratified by the IRLS risk-level in the TCGA-BLCA cohort. Yellow represents the expression of lncRNAs and levels of immune cells were upregulated while blue indicates downregulated. The relationship between IRLS risk-level and each clinicopathological characteristic were measured with the *χ*^*2*^ test. *p < 0.05, **p < 0.01, ***p < 0.001. **b** The immune cells network in TCGA-BLCA cohort. The colour of each cluster was: Cell cluster-A, blue; Cell cluster-B, yellow; Cell cluster-C, red; Cell cluster-D, brown. The size of circle indicated the statistical significant on OS for different immune cell type, which was shown as the formula log10 (Log-rank test p value). The lines connecting immune cells indicated cellular interactions. And the thickness of the line represents the correlation coefficient evaluated by spearman correlation analysis. Red represented positive correlation while blue represented negative correlation. **c** Correlation matrix of IRLS, immune-related lncRNAs and 24 types of immune cell. The blue indicated positive correlation and yellow indicated negative correlation. Shading colour and asterisks represents the value of corresponding correlation coefficients. *p < 0.05, **p < 0.01. **d** Violin plots demonstrated the correlation between IRLS with the levels of 24 types of immune cell. The p value was indicated in detail

As good representative of immune response status, IRLS might influence the TME immune cells infiltration in BLCA. In order to comprehensive characterize the landscape of TME immune cells infiltration in BLCA, ssGSEA was utilized to estimate the abundance of 24 immune cell types due to the specific reference gene sets mentioned above. The immune cells network, which could depict cellular interaction, cellular clusters and prognosis on the OS of BLCA patients, was established to exhibit the overall view of TME immune cells infiltration in BLCA (Fig. 7b). The results showed that there were four cellular clusters within 24 immune cell types, and the immunosuppressive cells such as Treg and NK CD56dim cells were highly correlated with all the other immune cell types. The KM survival curves showed that the innate immune cells (DC) and adaptive immune cells (cytotoxic cells, CD8 + T cells, T cells, T helper cells and Th17 cells) displayed beneficial effect, while innate immune cells (eosinophils, neutrophils, macrophages, mast cells and NK CD56dim cells) and adaptive immune cells (Tem cells, Th1 cells and Tgd cells) displayed harmful effect on the prognosis of BLCA patients (Additional file 10: Figure S6). The cluster heat map showed that the IRLS high-risk patients were filled with TME immune cells infiltration (Fig. 7a). Moreover, the spearman correlation analyses demonstrated that the IRLS lncRNAs were almost negative correlated with all the immune cells despite of NK CD56bright cells, which exerted a robustly anti-tumour effect (Fig. 7c). However, we also found that almost all the immune cells were filled in the IRLS high-risk group, indicating the poor prognosis might be relied on the immunosuppressive milieu (Fig. 7c, d).

### IRLS could predict the clinical response of immunotherapy

**Fig. 8:**
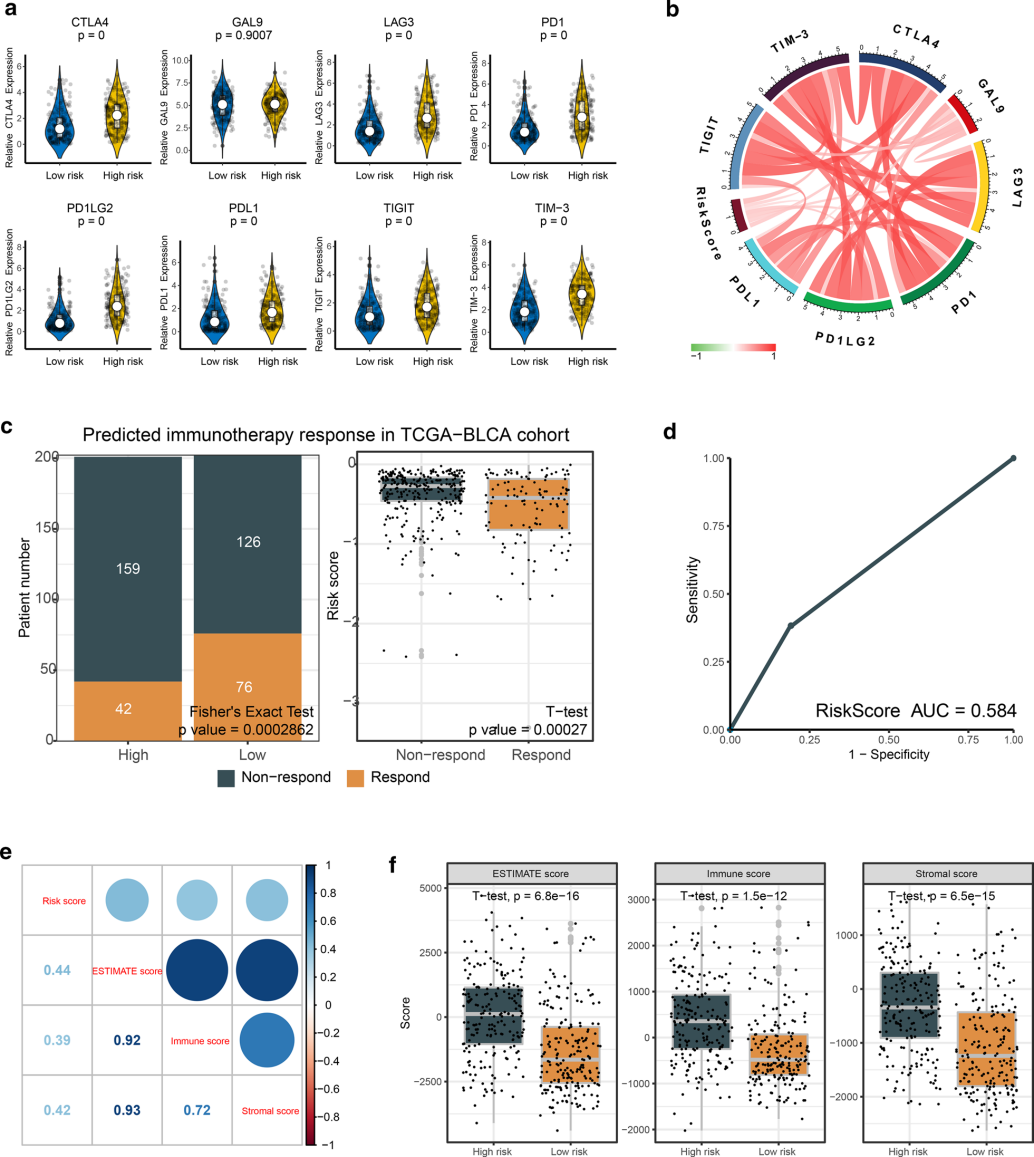
IRLS were efficient in prediction the immunotherapeutic benefit in BLCA. **a** Violin plots visualized the correlation between IRLS and immune-checkpoint-relevant genes, including CTLA-4, LAG-3, GAL9, PD-1, PD-1LG2, PD-L1, TIM-3 and TIGIT. **b** The correlation chord chart displayed the mutual correlation between IRLS and immune-checkpoint-relevant genes. **c** The distribution of immunotherapeutic response in indicated groups stratified by IRLS in TCGA-BLCA cohort based on the TIDE algorithm. Two-sided Fisher’s exact tests were used to analyse contingency tables for ICIs responder. **d** ROC curves for IRLS in predicting the immunotherapy response in the TCGA-BLCA cohort. **e** Correlation matrix of IRLS and immune scores, stromal scores and estimate score. **f** The box plot indicated the correlation between IRLS and immune scores, stromal scores and estimate score

Due to immunosuppressive atmosphere in IRLS high-risk patients, we next wonder to know whether there exited a correlation between IRLS and immune checkpoints such as PD-L1, which might trigger the immunosuppressive environment around the tumour and was reported to be predictive biomarkers for immunotherapy in multiple malignancies. Therefore, the routine immune-checkpoints, including CD274 (PD-L1), CTLA-4, LAG-3, LGALS9 (GAL9), HAVCR2 (TIM-3), PDCD1 (PD-1), PDCD1LG2 (PD-1LG2) and TIGHT, were selected to evaluate the relationship with IRLS. Then we found that the expression of almost all immune-checkpoints (CTLA-4, LAG-3, PD-1, PD-1LG2, PD-L1, TIM-3 and TIGIT) were upregulated in IRLS high-risk patients, which indicated the immunosuppressive TME and poor prognosis might be owed to the inducing of the immune checkpoints (Fig. 8a, b).

Recently, more and more study has reported the ICIs targeting immune-checkpoints such as PD-1 and PD-L1 could improve the efficiency in treatment of tumours. The TIDE algorithm, which was conducted to predict the immunotherapy responders through transcriptomic data, was utilized to explore whether IRLS could predict immunotherapeutic benefit in BLCA. The detailed output of TIDE algorithm in TCGA-BLCA cohort was shown in Additional file 11: Table S4. The result revealed that the number of immunotherapy responders were significantly higher in IRLS low-risk patients (76/202) compared with IRLS high-risk patients (42/201) (two-sided Fisher’s exact test, p = 0.0002862) (Fig. 8c). And the IRLS risk-score were robustly negative correlated with the immunotherapy response in BLCA patients (Fig. 8c). Moreover, the ROC curve also showed that IRLS displayed an appropriate predictive effect to ICIs response in TCGA-BLCA cohort (Fig. 8d). ESTIMATE algorithm, which could represent the tumour microenvironment (TME), was used to calculate the immune scores, stromal score and estimate scores in TCGA-BLCA cohort. We found that our IRLS also have a high positive correlation with these scores (Fig. 8e, f).

## Discussion

With the development of next generation sequencing, more and more transcriptomic data from the public database such as The Cancer Genome Atlas (TCGA) Research Network and Gene Expression Omnibus (GEO) could be easily obtained. By using the cohorts from the public database or own institute, many researchers found that non-coding RNAs (ncRNAs), such as microRNAs (miRNAs), long non-coding RNAs (lncRNAs), circular RNAs (circRNAs) etc. play vital roles in tumorigenesis by comparing cancer cells with corresponding pancanrerous cells [41]. In this context, analysis and evaluation of the genome and transcriptome sequencing data within a variety of human cancers from TCGA could provide us a comprehensive view on carcinogenesis according to aberrations at the genetic, epigenetic, and protein levels [42]. Despite genetic and epigenetic aberrations, such as methylation, histone modification, the dysregulated ncRNA expression has reported to be implicated in the function alterations and subsequent neoplasia induction [43]. Through mining the big data from the transcriptomic sequencing, many studies have demonstrated that lncRNAs acted as a key regulator for the development of cancer in various cellular functions, including proliferation, cell differentiation and DNA stability, etc. [44]. Moreover, whole genome sequencing analysis indicated that the most important mutation associated with tumorigenesis lied within the non-coding region of genome, which could alter the expression of lncRNAs [45]. All of these worked together to become a vicious circle for developing the malignancy.

As so importance in cancer development, recent years have showed a booming of lncRNAs in cancer research. A lot of lncRNAs, including MALAT1, HOTAIR, CCAT2, and AK126698, were found to be associated with non-small cell lung cancer (NSCLC) progression, metastasis, and invasion [46]. LncRNAs HULC and PCA-3 have been measured to be significantly upregulated in liver and prostate cancers, respectively [47]. Moreover, lncRNA urothelial carcinoma-associated 1 (UCA1), which was the most investigated lncRNA in BLCA, were involved in a variety of biological process in developing BLCA and might take responsibility for the drug resistance in BLCA [21, 22]. Despite focusing on the routine process in neoplasia, recent publications have seen a widespread alteration of lncRNAs in activation and priming of innate and adaptive immune response, such as T cell development, differentiation [48]. Conversely, researchers also found that these lncRNAs could regulate some important aspects of onco-immunity such as production of inflammatory mediators to form an immunosuppressive milieu through protein–protein interactions or capability to base-repair of RNA and DNA [49].

Through analysing the transcriptomic data via bioinformatics and machinery methods, many studies have already established the lncRNAs signature for predicting the prognosis of multiple cancers, including breast cancer [50], oesophageal cancer [51], as well as BLCA [24]. Furthermore, some even focused on investigating the immune-related lncRNAs signature in diffuse large B cell lymphoma [52], renal clear cell carcinoma [53], etc.

Here we aimed to construct an immune-related lncRNAs signature (IRLS) in BLCA, which is recognized an immunogenic cancer. Firstly, the M13664 (immune system process) and M19817 (immune response) gene sets, which were reported previously, have been used to represent the immune status of each sample in TCGA-BLCA cohort. By filtering the low expression lncRNAs, 101 immune-related lncRNAs were identified with significantly correlation with the immune status through estimation with ssGSEA. Kaplan-Meier (KM) survival analyses were used to measure the association between lncRNAs and prognosis in BLCA patients. We have found 702 lncRNAs significantly associated with the overall survival (OS) in patients with BLCA. Finally we have obtained 82 prognostic immune-related candidate lncRNAs in TCGA-BLCA cohort. Then we submitted them to LASSO cox regression analysis to establish a 5 immune-related lncRNAs signature (IRLS), which was capable for stratifying patients into the high-risk and low-risk groups with significantly different OS in TCGA-BLCA training cohort. The 5 lncRNAs (AC005014.2, AC010503.4, AL450384.2, LINC00930 and SH3BP5-AS1) were all protective factors for BLCA patients. Furthermore, qRT-PCR results validated that 5 lncRNAs were downregulated in BLCA cells and tissues compared with normal urothelium cells and pancanerous tissues respectively. Moreover, KM survival analyses from the testing and entire TCGA-BLCA cohort also suggested that IRLS has good reproducibility and are robustness in prognosis prediction for BLCA patients. We also found that IRLS was vigorously positive correlated with the malignancy clinical features such as high TNM stage, non-papillary in BLCA. As a heterogeneous disease with so many clinicopathological characteristics and risk factors, the stratification analyses should be utilized to figure out whether our signature was independence of them. The results demonstrated that IRLS could clearly distinguish patients with all subgroups. Additionally, IRLS remained as an independent prognostic factor by combination with other risk factors through univariate and multivariate Cox regression analyses. Within the nomogram integrating independent prognostic factors, IRLS contributed more and performs better in survival predicting. All of these suggested that IRLS acted as an oncogenic role and was able to improve prognosis accuracy in BLCA.

Interestingly, the function and annotation analyses demonstrated that the gene sets within immune response related pathway, such as inflammatory response, complement, IL2-STAT5 signalling, etc. were significantly positive correlated with each other, which again emphasized the importance of immune response regulation in BLCA. The results of GSVA revealed that the angiogenesis related pathway, immune response related pathway and EMT related pathway, etc. which are considered immunosuppressive and play a vital role in tumorigenesis, were enriched in IRLS high-risk group. Furthermore, the GSEA also showed the positive correlation between IRLS risk-score and M13664 (immune system process) and M19817 (immune response) gene sets. The functional annotation was entirely consistent with the survival analyses and indicated the IRLS was a good representative for immune response status in BLCA.

TME immune cells infiltration in situ now has been recognized as critical invaluable information for predicting the prognosis and immunotherapy response in various cancers according to the clinical trials with ICIs [54, 55]. So we made a comprehensive analysis of the TME immune cells infiltration landscape by estimation the abundance of 24 immune cell types in BLCA. Strikingly, we found that the IRLS high-risk group were filled with all types of immune cells, especially immunosuppressive cells such as Treg, macrophages and NK CD56dim cells, which could formed the immunosuppressive atmosphere to hamper the activation of CD8 + T cells and NK CD56bright cells for eradicating the tumour cells [56, 57]. By clearly analyse the immune cells network, we were amazed to find that Treg and NK CD56dim cells were positive associated with all immune cells, no matter protective or harmful. So it was proposed that there existed a negative regulation system, which immunosuppressive cells will response to changes of other immune cells and dominate the central position within the tumour immune microenvironment in BLCA. To this effect, we inferred that the poor prognosis of IRLS high-risk patients might relay on this tumour immunosuppressive microenvironment. And the results from the ESTIMATE algorithm indicated IRLS was positive associated with the immune scores, stromal scores and estimate scores, which could represent the tumour microenvironment. Moreover, the immune-checkpoints, such as CTLA-4, PD-1/PD-L1, etc., also act as rheostat in immune response regulation by inhibiting the priming of protective immune cells and immune surveillance [58, 59]. Therefore, we found that the expression of immune checkpoints increased in IRLS high-risk patients, prompting us to measure its potential in ICIs response prediction. Encouragingly, with the help of TIDE algorithm, IRLS was proved to be efficiency in predicting the immunotherapy response in TCGA-BLCA cohort. Therefore, IRLS was robustly negative correlated with the immunotherapy response and there were more immunotherapeutic responders in IRLS low-risk groups (76/202) than high-risk groups (42/201). All of these indicated that IRLS was a potent biomarker for predicting the immunotherapy response.

## Conclusions

We have made a comprehensive estimation of immune-related lncRNAs and established a prognostic and predictive IRLS for response to ICIs in BLCA, which has broaden our eyes in immunotherapies and may provide a useful scoring system for clinical utility.

## Supplementary information

**Additional file 1.** Ethics Committee Approval (number: 2015029).

**Additional file 2: Table S1.** List of primers for qRT-PCR.

**Additional file 3: Figure S1.** A flow diagram and design of the study.

**Additional file 4: Figure S2.** Establishment of the most valuable prognostic immune-related lncRNAs signature (IRLS) through LASSO Cox regression model.

**Additional file 5: Figure S3.** The prognosis effect of 5 immune-related lncRNA within IRLS.

**Additional file 6: Figure S4.** Association between the IRLS and clinicopathological characteristics.

**Additional file 7: Table S2.** Summary of GSVA for hallmark gene sets in TCGA-BLCA cohort.

**Additional file 8: Table S3.** Summary of ssGSEA scores for hallmark gene sets in TCGA-BLCA cohort.

**Additional file 9: Figure S5.** The GSEA plot of M13664 (immune system process) and M19817 (immune response) gene sets in TCGA-BLCA training (A), testing (B) and entire cohort (C).

**Additional file 10: Figure S6.** The KM survival analyses of each immune cell types in TCGA-BLCA cohort.

**Additional file 11: Table S4.** The detailed information of immunotherapy response based on TIDE algorithm in TCGA-BLCA cohort.

## Data Availability

All data generated or analysed during this study are included in this published article and its Additional files.

## Abbreviations

aDCs: Activated DCs
AUC: Area under curve
BCG: Bacillus Calmette-Guerin
BLCA: Bladder cancer
CI: Confidence interval
circRNA: Circular RNA
DC: Dendritic cells
DCA: Decision curve analysis
CTLA-4: Cytotoxic T-lymphocyte-associated protein 4
EMT: Epithelial-mesenchymal transition
ESTIMATE: Estimation of STromal and Immune cells in MAlignant Tumours using Expression data
FDA: Food and Drug Administration
GAL9: LGALS9
GEO: Gene Expression Omnibus
GSEA: Gene Set Enrichment Analysis
GSVA: Gene Set Variation Analysis
HE: Hematoxylin and eosin
HR: Hazard ratio
ICIs: Immune-checkpoint inhibitors
IRLS: Immune-related lncRNAs signature
KM: Kaplan–Meier
LASSO: Least absolute shrinkage and selection operator
MIBC: Muscle-invasive bladder cancer
miRNAs: microRNA
NES: Normalized enrichment score
NKs: Natural killer cells
NMIBC: Non-muscle-invasive bladder cancer
NSCLC: Non-small cell lung cancer
OS: Overall survival
pDCs: Plasmacytoid DC
PD-1: Programmed death-1
PD-L1: Programmed death-ligand-1
ROC: Receiver operating characteristic curve
ssGSEA: Single-sample gene set enrichment analysis
TCGA: The Cancer Genome Atlas
Tcm: T central memory
Tem: T effector memory
Th1: T helper 1
Th2: T helper 2
Tim-3: Havcr2
TNM: Tumour Node Metastasis
TME: Tumour microenvironment
Treg: Regulatory T cells
Tγδ: T follicular helper
