# Supplementary material for: Immune-related long non-coding RNA signature identified prognosis and immunotherapeutic efficiency in bladder cancer (BLCA)
Source: Cancer Cell Int. 2020 Jun 26;20:276. doi: 10.1186/s12935-020-01362-0 (PMC7320553; doi:10.1186/s12935-020-01362-0)
Supplement: Supplementary file 2 — Additional file 2: Table S1. List of primers for qRT-PCR. [file 12935_2020_1362_MOESM2_ESM.docx]

**Additional file 2: Table S1.** List of primers for qRT-PCR.

| **Symbol** | **Forward primer (5’-3’)** | **Reverse primer (5’-3’)** | **Annealing Temperature ( °C)** |
| --- | --- | --- | --- |
| *AC005014.2* | 5'- GCACAACTGCCCTTGGGATAA -3' | 5'- CTGGGGCTTTGGAATCCAGC -3' | 60 |
| *AC010503.4* | 5'- GAGGCAGTCATCCCCAAAGA -3' | 5'- GCTCAAAATTGTCCGTGCGA -3' | 60 |
| *AL450384.2* | 5'- AGGATTCAGGGCATGGGAAGT -3' | 5'- CAGCAGGTTCACTGTCTGTCA -3' | 60 |
| *LINC00930* | 5'- ACCCCCAAGGAAGAGTCAGT -3' | 5'- ACATGTCAGAAGCCGTCAGG -3' | 60 |
| *SH3BP5-AS1* | 5'- ACCCTGCTAGCCTTAAGATGC -3' | 5'- GAGGTTGTCACCTGGATGGG -3' | 60 |
