# Supplementary figures and images for: Immune-related long non-coding RNA signature identified prognosis and immunotherapeutic efficiency in bladder cancer (BLCA)
Source: Cancer Cell Int. 2020 Jun 26;20:276. doi: 10.1186/s12935-020-01362-0 (PMC7320553; doi:10.1186/s12935-020-01362-0)

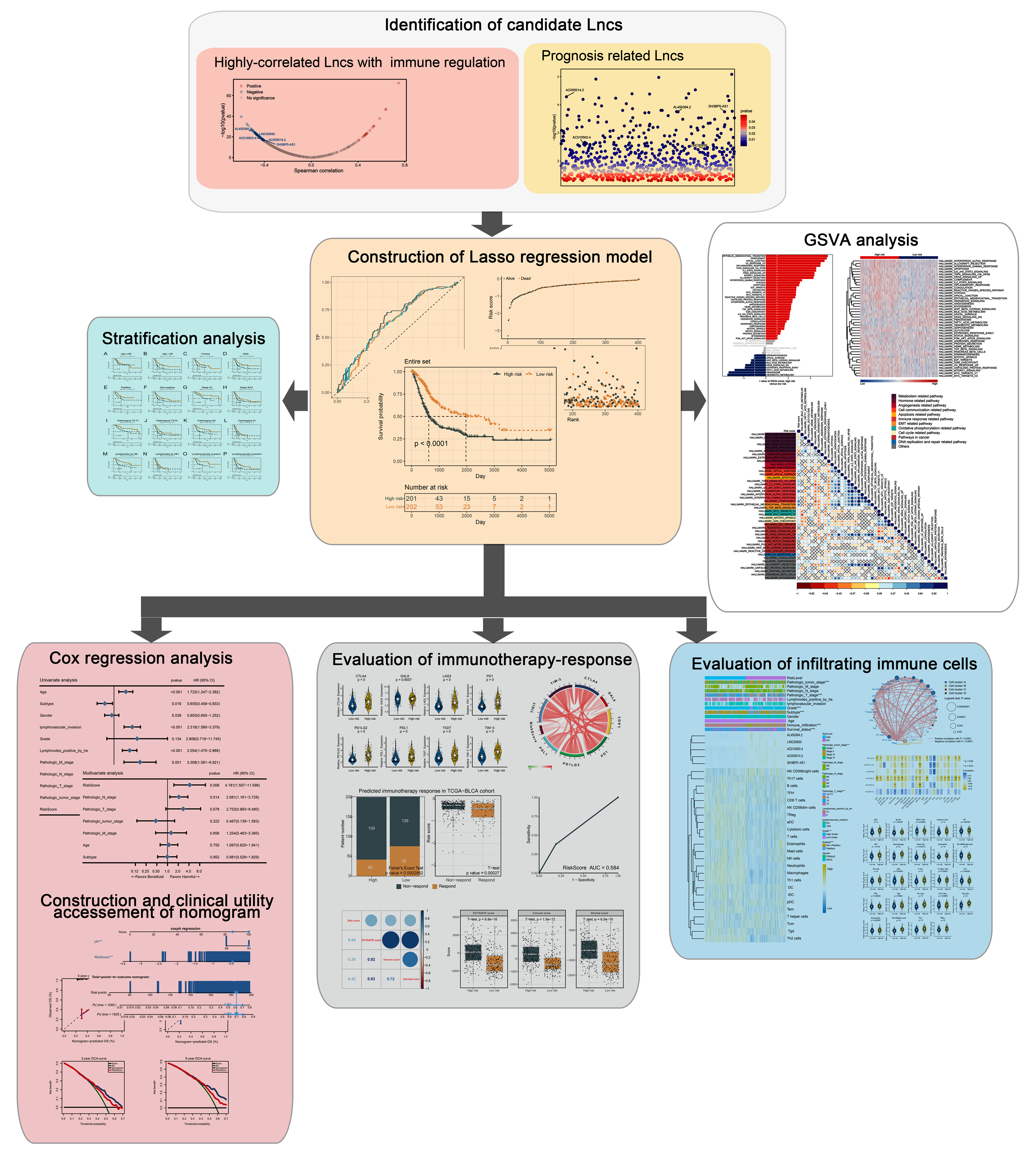

Supplement: Supplementary file 3 — Additional file 3: Figure S1. A flow diagram and design of the study. [file 12935_2020_1362_MOESM3_ESM.tif]

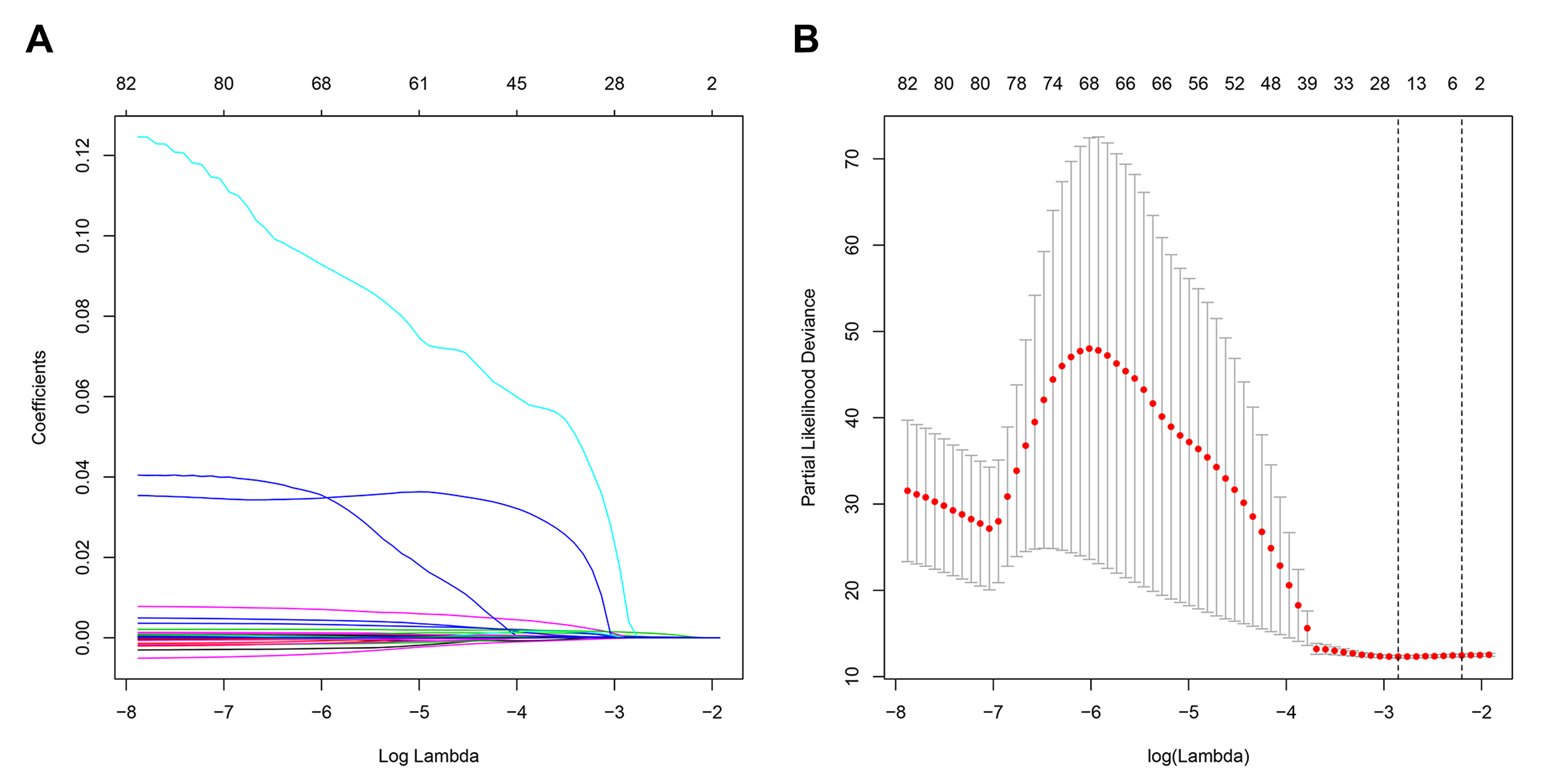

Supplement: Supplementary file 4 — Additional file 4: Figure S2. Establishment of the most valuable prognostic immune-related lncRNAs signature (IRLS) through LASSO Cox regression model. [file 12935_2020_1362_MOESM4_ESM.tif]

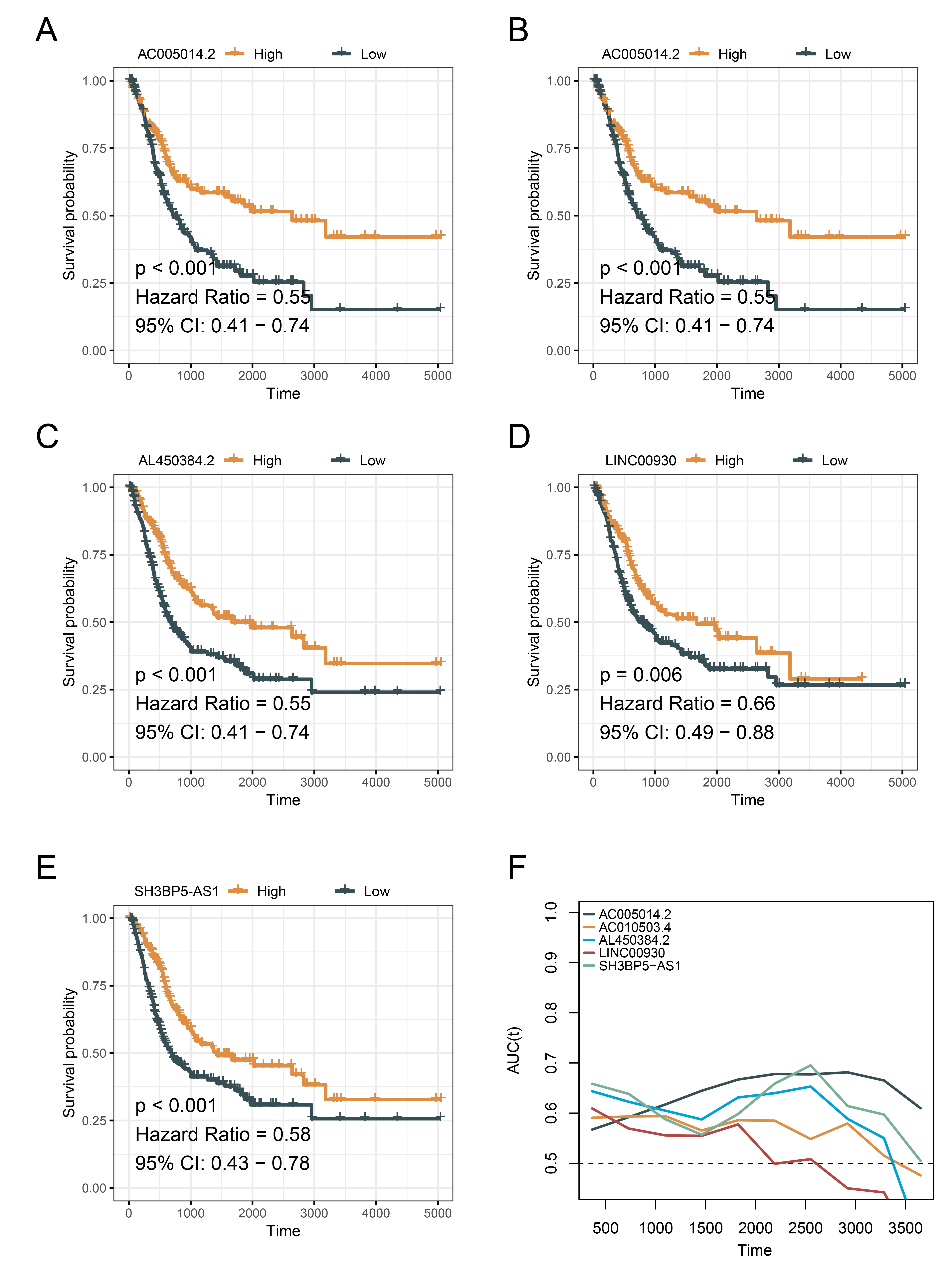

Supplement: Supplementary file 5 — Additional file 5: Figure S3. The prognosis effect of 5 immune-related lncRNA within IRLS. [file 12935_2020_1362_MOESM5_ESM.tif]

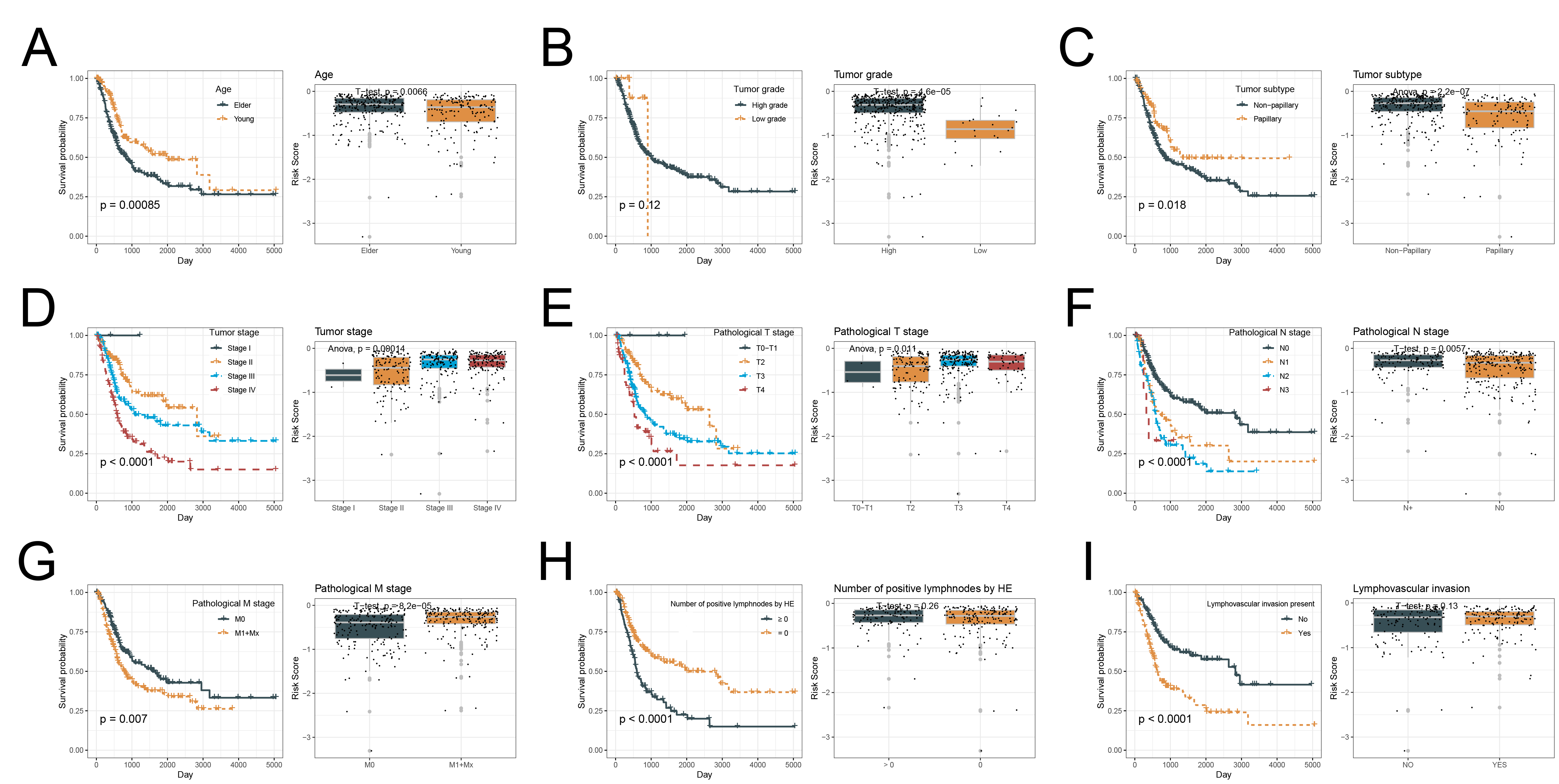

Supplement: Supplementary file 6 — Additional file 6: Figure S4. Association between the IRLS and clinicopathological characteristics. [file 12935_2020_1362_MOESM6_ESM.tif]

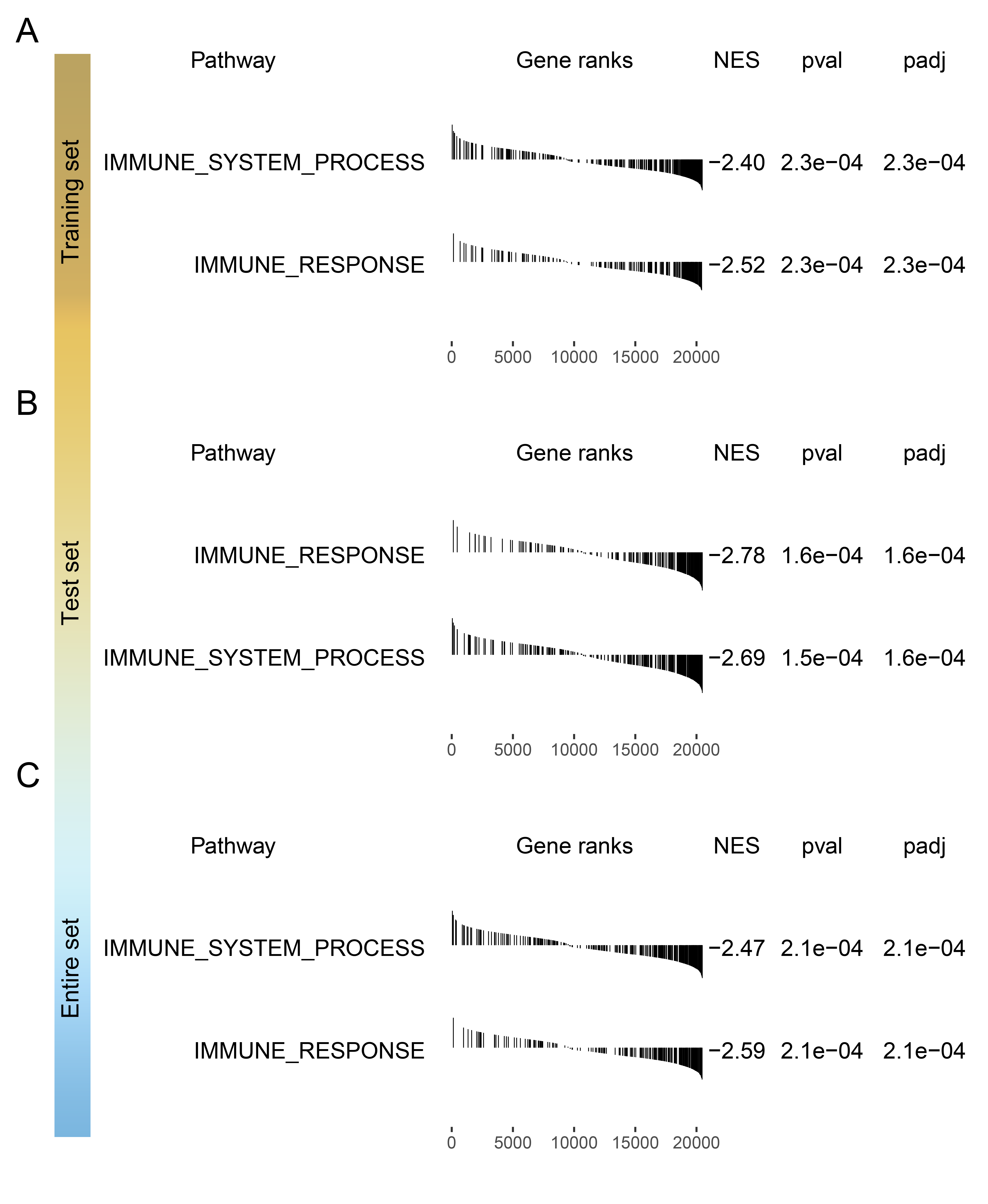

Supplement: Supplementary file 9 — Additional file 9: Figure S5. The GSEA plot of M13664 (immune system process) and M19817 (immune response) gene sets in TCGA-BLCA training (A), testing (B) and entire cohort (C). [file 12935_2020_1362_MOESM9_ESM.tif]

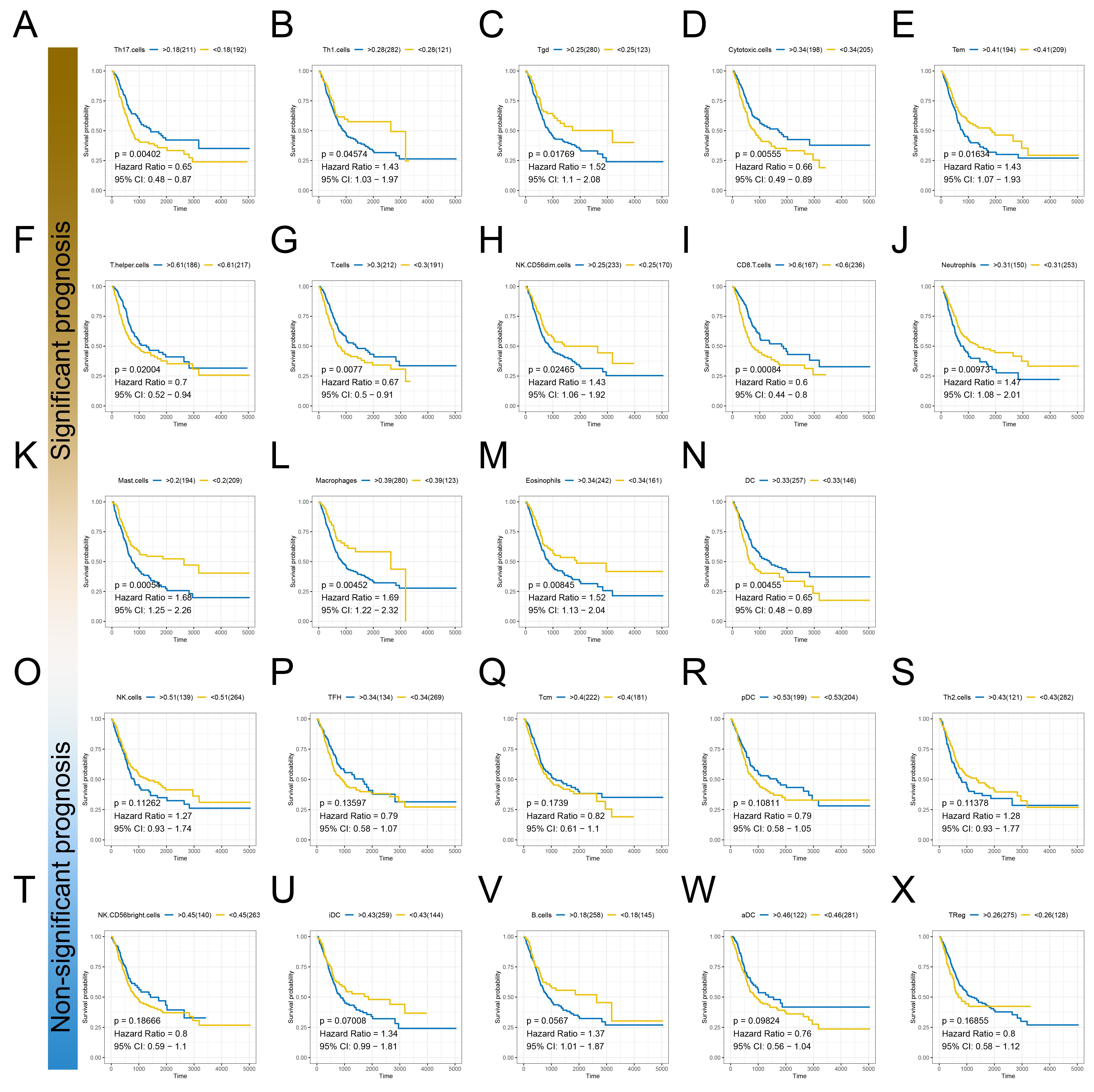

Supplement: Supplementary file 10 — Additional file 10: Figure S6. The KM survival analyses of each immune cell types in TCGA-BLCA cohort. [file 12935_2020_1362_MOESM10_ESM.tif]
