# Supplementary material for: Immune-related long non-coding RNA signature identified prognosis and immunotherapeutic efficiency in bladder cancer (BLCA)
Source: Cancer Cell Int. 2020 Jun 26;20:276. doi: 10.1186/s12935-020-01362-0 (PMC7320553; doi:10.1186/s12935-020-01362-0)
Supplement: Supplementary file 7 — Additional file 7: Table S2. Summary of GSVA for hallmark gene sets in TCGA-BLCA cohort. [file 12935_2020_1362_MOESM7_ESM.docx]

**Additional file 7: Table S2 Summary of GSVA for hallmark gene sets in TCGA-BLCA cohort.**

| **TCGA-BLCA** | **logFC** | **AveExpr** | **t-value** | **P.Value** | **adj.P.Val** |
| --- | --- | --- | --- | --- | --- |
| HALLMARK_EPITHELIAL_MESENCHYMAL_TRANSITION | 0.40095241 | 0.000187596 | 11.09716193 | 3.52E-25 | 1.76E-23 |
| HALLMARK_COMPLEMENT | 0.224905168 | -0.00923024 | 10.90404898 | 1.84E-24 | 4.59E-23 |
| HALLMARK_APICAL_JUNCTION | 0.18575412 | -0.015336403 | 10.50846048 | 5.14E-23 | 8.57E-22 |
| HALLMARK_UV_RESPONSE_UP | 0.135913469 | -0.022230477 | 9.823496752 | 1.40E-20 | 1.75E-19 |
| HALLMARK_INFLAMMATORY_RESPONSE | 0.2961915 | -0.003109499 | 9.752078623 | 2.48E-20 | 2.48E-19 |
| HALLMARK_TNFA_SIGNALING_VIA_NFKB | 0.247838398 | -0.010247325 | 9.021458693 | 7.40E-18 | 6.16E-17 |
| HALLMARK_IL2_STAT5_SIGNALING | 0.187200634 | -0.014145201 | 9.000811376 | 8.65E-18 | 6.18E-17 |
| HALLMARK_KRAS_SIGNALING_UP | 0.165229303 | -0.004203072 | 8.718004598 | 7.26E-17 | 4.54E-16 |
| HALLMARK_MTORC1_SIGNALING | 0.234008071 | -0.023859016 | 8.686981753 | 9.14E-17 | 5.08E-16 |
| HALLMARK_ALLOGRAFT_REJECTION | 0.27468534 | 0.001119671 | 8.533579201 | 2.84E-16 | 1.42E-15 |
| HALLMARK_XENOBIOTIC_METABOLISM | -0.113107652 | -0.002837762 | -6.884367225 | 2.21E-11 | 1.00E-10 |
| HALLMARK_PEROXISOME | -0.136090979 | -0.01447638 | -6.677660572 | 7.98E-11 | 3.32E-10 |
| HALLMARK_INTERFERON_GAMMA_RESPONSE | 0.219628119 | -0.011759766 | 6.485141254 | 2.57E-10 | 9.89E-10 |
| HALLMARK_HYPOXIA | 0.103420342 | -0.001034346 | 6.098001823 | 2.50E-09 | 8.92E-09 |
| HALLMARK_APOPTOSIS | 0.100046345 | -0.0092506 | 5.95371735 | 5.66E-09 | 1.89E-08 |
| HALLMARK_FATTY_ACID_METABOLISM | -0.103016376 | 0.000333416 | -5.585573479 | 4.27E-08 | 1.33E-07 |
| HALLMARK_E2F_TARGETS | 0.205700346 | -0.012412576 | 5.464494523 | 8.10E-08 | 2.38E-07 |
| HALLMARK_GLYCOLYSIS | 0.094155962 | -0.026185976 | 5.448005528 | 8.83E-08 | 2.45E-07 |
| HALLMARK_MYC_TARGETS_V2 | 0.209604149 | -0.027527024 | 5.381440412 | 1.25E-07 | 3.13E-07 |
| HALLMARK_MYC_TARGETS_V1 | 0.18046741 | -0.021501923 | 5.381182803 | 1.25E-07 | 3.13E-07 |
| HALLMARK_REACTIVE_OXIGEN_SPECIES_PATHWAY | 0.13812608 | -0.024830369 | 5.27247197 | 2.19E-07 | 5.21E-07 |
| HALLMARK_UNFOLDED_PROTEIN_RESPONSE | 0.092787917 | -0.02760288 | 4.791823412 | 2.32E-06 | 5.27E-06 |
| HALLMARK_INTERFERON_ALPHA_RESPONSE | 0.179853411 | -0.016768286 | 4.738961748 | 2.97E-06 | 6.46E-06 |
| HALLMARK_ANGIOGENESIS | 0.157412071 | 0.016999161 | 4.557008828 | 6.87E-06 | 1.43E-05 |
| HALLMARK_HEME_METABOLISM | 0.056983755 | -0.003546142 | 4.508835817 | 8.53E-06 | 1.71E-05 |
| HALLMARK_TGF_BETA_SIGNALING | 0.113570642 | -0.014905065 | 4.495150076 | 9.07E-06 | 1.75E-05 |
| HALLMARK_G2M_CHECKPOINT | 0.134341903 | -0.022391677 | 4.335563919 | 1.83E-05 | 3.40E-05 |
| HALLMARK_IL6_JAK_STAT3_SIGNALING | 0.113462044 | 0.009031284 | 4.283040778 | 2.30E-05 | 4.11E-05 |
| HALLMARK_PANCREAS_BETA_CELLS | 0.08549194 | 0.068961846 | 4.237907112 | 2.79E-05 | 4.82E-05 |
| HALLMARK_HEDGEHOG_SIGNALING | 0.122678105 | -0.007290983 | 4.154513366 | 3.97E-05 | 6.62E-05 |
| HALLMARK_ESTROGEN_RESPONSE_EARLY | -0.065364442 | -0.014514188 | -3.760003361 | 0.000194784 | 0.000314167 |
| HALLMARK_COAGULATION | 0.075329226 | 0.031591591 | 3.700153607 | 0.000245067 | 0.000382917 |
| HALLMARK_KRAS_SIGNALING_DN | -0.044941925 | 0.022647698 | -3.688545556 | 0.000256138 | 0.000388088 |
| HALLMARK_ANDROGEN_RESPONSE | 0.060174811 | -0.010030004 | 3.433327069 | 0.00065735 | 0.000966691 |
| HALLMARK_ADIPOGENESIS | 0.05316295 | -0.010573664 | 3.352077714 | 0.000877061 | 0.001252944 |
| HALLMARK_MITOTIC_SPINDLE | 0.06037246 | -0.021528161 | 3.298508965 | 0.001057431 | 0.001468654 |
| HALLMARK_BILE_ACID_METABOLISM | -0.046170744 | 0.004689828 | -2.751541681 | 0.006196432 | 0.008373557 |
| HALLMARK_NOTCH_SIGNALING | 0.068650409 | -0.012762823 | 2.687816744 | 0.007486999 | 0.009851314 |
| HALLMARK_WNT_BETA_CATENIN_SIGNALING | -0.057930496 | -0.022204174 | -2.628309605 | 0.008905509 | 0.01141732 |
| HALLMARK_PROTEIN_SECRETION | 0.061965951 | -0.014359994 | 2.586259798 | 0.010048536 | 0.01256067 |
| HALLMARK_APICAL_SURFACE | -0.041544191 | -0.011661897 | -2.139092206 | 0.033022334 | 0.040271139 |
| HALLMARK_MYOGENESIS | 0.054016951 | 0.009492187 | 2.120946191 | 0.034530066 | 0.041107222 |
| HALLMARK_SPERMATOGENESIS | -0.030400721 | 0.021771282 | -2.107019952 | 0.035726864 | 0.041542865 |
| HALLMARK_PI3K_AKT_MTOR_SIGNALING | 0.031395163 | -0.020619956 | 2.08815404 | 0.037404679 | 0.042505317 |
| HALLMARK_UV_RESPONSE_DN | 0.037611833 | -0.016353667 | 1.634219255 | 0.102985615 | 0.114428461 |
| HALLMARK_OXIDATIVE_PHOSPHORYLATION | 0.042797217 | -0.033264336 | 1.290940902 | 0.197456553 | 0.214626688 |
| HALLMARK_P53_PATHWAY | -0.014115213 | -0.022558439 | -0.66935965 | 0.503645401 | 0.53579298 |
| HALLMARK_DNA_REPAIR | -0.009721115 | -0.031484482 | -0.458862954 | 0.646577396 | 0.673518121 |
| HALLMARK_ESTROGEN_RESPONSE_LATE | -0.006678581 | -0.01988449 | -0.363555201 | 0.716378846 | 0.730998823 |
| HALLMARK_CHOLESTEROL_HOMEOSTASIS | 0.000804512 | -0.014840728 | 0.037547024 | 0.970067246 | 0.970067246 |
